# On Aerial Robots with Grasping and Perching Capabilities: A Comprehensive Review

**DOI:** 10.3389/frobt.2021.739173

**Published:** 2022-03-25

**Authors:** Jiawei Meng, Joao Buzzatto, Yuanchang Liu, Minas Liarokapis

**Affiliations:** ^1^ Department of Mechanical Engineering, University College London, London, United Kingdom; ^2^ New Dexterity Research Group, Department of Mechanical and Mechatronics Engineering, The University of Auckland, Auckland, New Zealand

**Keywords:** unmanned aerial vehicles, aerial robots, grasping, perching, robotic gripping mechanisms

## Abstract

Over the last decade, there has been an increased interest in developing aerial robotic platforms that exhibit grasping and perching capabilities not only within the research community but also in companies across different industry sectors. Aerial robots range from standard multicopter vehicles/drones, to autonomous helicopters, and fixed-wing or hybrid devices. Such devices rely on a range of different solutions for achieving grasping and perching. These solutions can be classified as: 1) simple gripper systems, 2) arm-gripper systems, 3) tethered gripping mechanisms, 4) reconfigurable robot frames, 5) adhesion solutions, and 6) embedment solutions. Grasping and perching are two crucial capabilities that allow aerial robots to interact with the environment and execute a plethora of complex tasks, facilitating new applications that range from autonomous package delivery and search and rescue to autonomous inspection of dangerous or remote environments. In this review paper, we present the state-of-the-art in aerial grasping and perching mechanisms and we provide a comprehensive comparison of their characteristics. Furthermore, we analyze these mechanisms by comparing the advantages and disadvantages of the proposed technologies and we summarize the significant achievements in these two research topics. Finally, we conclude the review by suggesting a series of potential future research directions that we believe that are promising.

## 1 Introduction

Aerial robots exhibit increased mobility compared to ground robots as they are not restricted by terrains and they can navigate hard-to-access locations (Ruggiero et al., 2018). The multicopter drone is the most representative category of aerial robots, and several drone production companies have emerged during the last decade, including but not limited to: 1) Parrot which is based in the European Union (Parrot, 2019), 2) DJI which is based in China (DJI, 2019), and 3) Prodrone which is based in Japan (Prodrone, 2019). The remarkable progress made in advancing the technologies of aerial robots and decreasing the development costs has led to a profound impact and widespread use of multicopter devices by the military, various industries, as well as by consumers in various applications:

1. photography and inspection (Glade, 2000; Daniel et al., 2009; Morgan et al., 2010; Irizarry et al., 2012; Nikolic et al., 2013; Bemis et al., 2014; Colomina and Molina, 2014; Lucieer et al., 2014; Restas, 2015; Sa et al., 2015; Tang and Shao, 2015; Panagiotidis et al., 2017; Thiel and Schmullius, 2017; Cliff et al., 2018; Hang et al., 2019).

2. transportation (Thiels et al., 2015; Amazon Prime Air, 2019; DHL-Parcelcopter, 2019; Heutger and Kückelhaus, 2014; X-Wing, 2019; Flirtey, 2019; Momont, 2016; United Parcel Service Drone Florida Package Delivery, 2019; Zipline, 2019; Uber-AIR, 2019; Antwork-Robtics, 2019; SARAH Hand from Laval University, 2019; Gosselin and Laliberte, 2010; Lester et al., 2017; Chien and Wagstaff, 2017).

3. architecture, building, and construction (Lindsey et al., 2011; Willmann et al., 2012; Hunt et al., 2014; Werfel et al., 2014; Keating et al., 2017; Pawar et al., 2017; Zhang G. et al., 2019).

**FIGURE 1:**
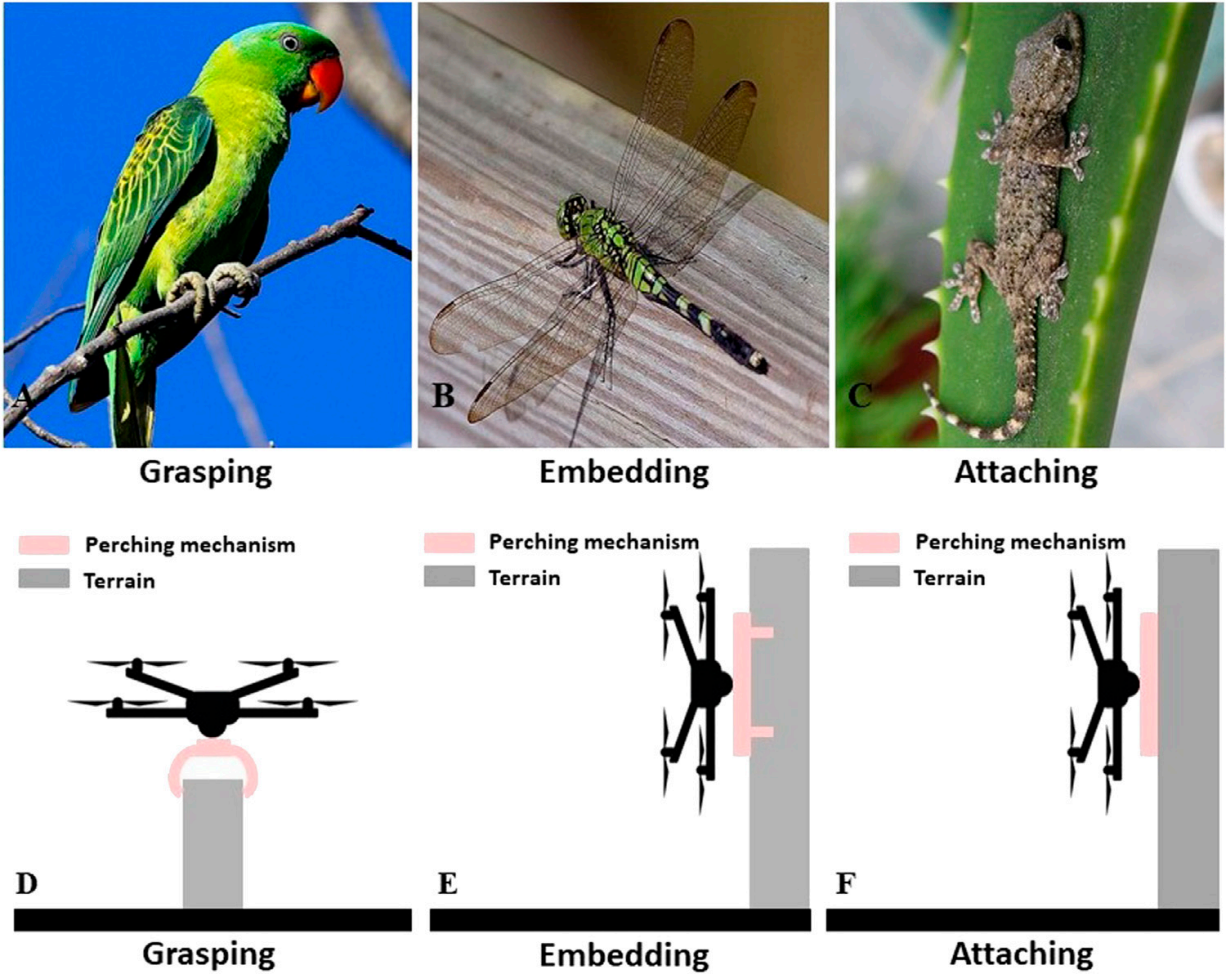
Perching behaviours of different creatures in nature and aerial robots with different perching mechanisms: **(A)** demonstrates a parrot perching on a tree branch based on the grasping capability of its claws; **(B)** demonstrates a dragonfly perching on a tree trunk based on the embedding capability of the spines on its feet; **(C)** demonstrates a gecko perching on a glass based on the attaching capability of its feet; **(D)** demonstrates an aerial robot perching on a pole by grasping mechanism; **(E)** demonstrates an aerial robot perching on a wall by embedding mechanism, and **(F)** demonstrates an aerial robot perching on a wall by attaching mechanism.

**FIGURE 2:**
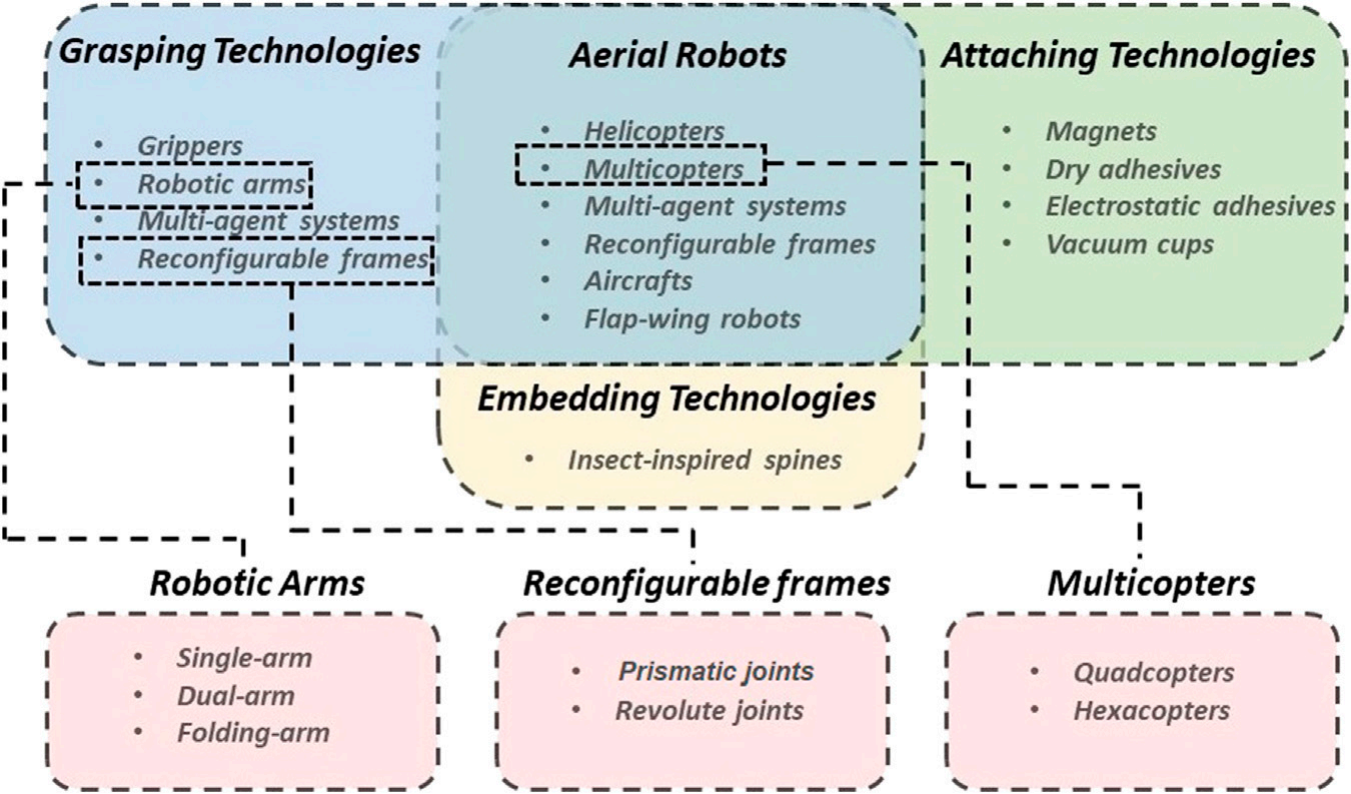
A comparison of aerial robots with different perching technologies. As demonstrated in this figure, grasping technologies are highlighted in the light blue part, attaching technologies are highlighted in the light green part, and embedding technologies are highlighted in the light yellow part. Furthermore, it is worth noting that robotic arm in the grasping technologies section includes three sub-categories: single-arm, dual-arm, and folding-arm; multicopter in the aerial robots section includes two sub-categories: quadcopter and hexacopter; reconfigurable frame in the grasping technologies section includes two sub-categories: prismatic joint and revolute joint.

In the last 2 decades, the field of aerial robots has evolved considerably with significant contributions both in software and hardware. While there are some reviews that focus on the state-of-art in control and modeling for aerial manipulation mechanisms (Ruggiero et al., 2018; Meng et al., 2020), there is still a lack of a comprehensive review focusing on the hardware and design innovations for aerial grasping and perching. Grasping and perching capabilities are two crucial characteristics of aerial robots that allow them to efficiently execute a plethora of tasks and interact with unstructured and dynamic environments. Grasping and perching impact the stability, load capability, control and planning complexity, and overall performance of aerial robots. A comparison of the perching behaviors and capabilities of different creatures in nature as well as of aerial robots that are equipped with different perching mechanisms is shown in Figure 1. Another comparison of different perching technologies of aerial robots is shown in Figure 2. Grasping is defined as an act that consists of three different steps: 1) reaching a target object, 2) establishing contact with the object surface, and 3) securing and holding the object firmly. Thus, grasping is a fundamental step of aerial perching and aerial transportation. Both aerial perching and aerial transportation require an increased grasping capability. Aerial grasping can be achieved by: 1) a single aerial robot equipped with a simple gripper, a robotic arm and a gripper, or a reconfigurable frame or 2) a multi-agent system/swarm that synergistically grasps an object. On the other hand, perching is defined as the process of supporting the aerial robot’s weight using grasping, attaching, or embedding solutions (Hang et al., 2019). Hence grasping is one of the candidate approaches for achieving perching. Grasping-based perching can be achieved by a simple gripper or an arm-gripper system. Embedding-based perching behaviour can be achieved by insect-inspired spines. Attaching-based perching behaviour can be achieved by magnets, dry adhesives, electrostatic adhesives, and vacuum cups on planar terrains such as a ceiling or a wall. Numerous examples of perching can be found in nature, including but not restricted to perching executed birds and flying insects, as shown in Figure 1.

In this paper, we focus on reviewing and comparing the innovative ideas that have been proposed for the mechanical design of aerial robots with grasping and perching capabilities. A systematic literature review approach was used, considering all relevant works that were retrieved using a plethora of keywords including combinations of the keywords “aerial”, “UAV”, “grasping”, “gripping”, “manipulation”, and “perching” in several databases including IEEEXplore, Web of Science, and Google Scholar. Furthermore, this review prioritized recent works that proposed original concepts for aerial grasping and perching. Related works that describe existing mechanisms that are not proposed for the first time are excluded from the conducted analysis. Furthermore, this review highlights the research groups and institutions developing these novel technologies to help other researchers better identify the key developments and researchers in the field. Finally, a comparison of all the discussed technologies is presented in Section 5. Overall, this work attempts to provide an in-depth and comprehensive review for researchers interested in developing novel aerial robots with grasping and perching capabilities.

The rest of this review paper is organized as follows: Section 2 describes the principles of grasping and perching, illustrating and analyzing a series of mechanisms developed for aerial grasping as well as for the development of grasping capable aerial robots, Section 3 illustrates and analyzes a series of perching capable aerial robots, Section 4 compares the current technologies used for aerial grasping and perching and then presents the major achievements in these fields, while Section 5 concludes the paper and Section 6 provides some insight into possible future trends for these two research areas.

A comprehensive list of the terms and notions discussed in this work can be found in the glossary presented in Table 1.

**TABLE 1:** Glossary - A list of comprehensive descriptions of the notions used in this review.

Glossary name	Explanation
Adaptive/compliant	Adaptive/compliant in the context of grasping means the mechanism can conform to the object shape using underactuation and post-contact reconfiguration and/or structural compliance. Hence a mechanism with this property can provide robust grasping.
Center of gravity	Center of gravity (CoG) of is the point which the whole body’s mass can be assumed to be concentrated at.
Degrees of freedom	Degrees of freedom (DoF) is the number of independent motions that the joints of a robot can execute. In the
	case of a mechanism made of several bodies, the number of possible independent relative motions between
	the pieces/joints of the mechanism.
Dexterity	The dexterity of a robot hand/gripper can be defined as the ability to grasp and manipulate objects or the environment with skillfulness. It refers to how gracefully the robot hand/gripper can interact and handle objects and take any necessary actions on the objects.
End-effector	End-effector is the device at the end of a robotic arm such as a robotic gripper that facilitates grasping of objects or interaction with the environment.
Fully-actuated	Fully-actuated means the number of DoFs of the mechanism is equal to the number of the used actuators. Hence
	fully-actuated mechanism is typically highly skillful and capable to perform complex in-hand manipulation.
Multi-agent	A multi-agent system is a system composed of multiple interacting intelligent agents.
Routh–Hurwitz criterion	Routh–Hurwitz criterion is a mathematical test, which is a necessary and sufficient condition to ensure
	the stability of a linear time-invariant control systems.
Tendon-driven	Tendon-driven means that multiple joints of a mechanism are driven simultaneously by employing a wire/tendon
	that passes through the mechanism, offering motion transmission.
Thrust-to-weight ratio	Thrust-to-weight ratio (T/W ratio) is a measurement of the total thrust produced by the aerial robot to its weight.
Underactuated	Underactuated means that the number of DoFs of the mechanism is more than the number of the used actuators.
	Hence an underactuated mechanism is relatively lightweight, low-cost, and efficient compared to full-actuated
	mechanisms.

## 2 Aerial Grasping and Manipulation Mechanisms

This section presents and discusses the research conducted over the years on aerial grippers and aerial robots equipped with grasping and manipulation mechanisms.

### 2.1 Aerial Grasping Mechanisms

Mounting a gripper onto an aerial vehicle is the most intuitive and straightforward way to achieve aerial grasping. Therefore, this and the following subsections focus on grippers and manipulators that can be used to perform aerial grasping. Such devices should be portable, lightweight, and able to be mounted on different types of aerial platforms.

#### 2.1.1 GRAB Lab, Yale University

**FIGURE 3:**
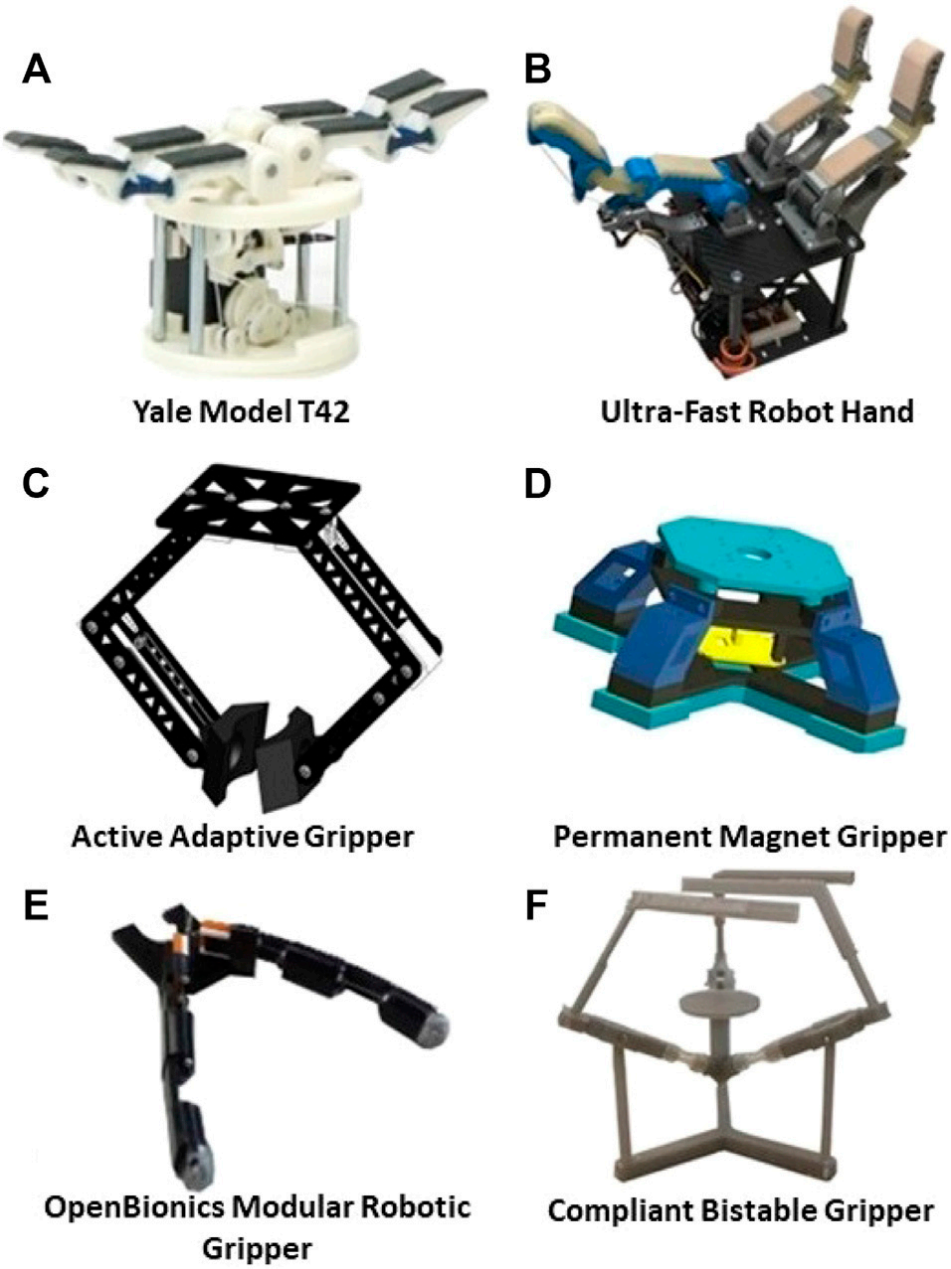
Grippers for aerial grasping: **(A)** is the Yale Model T42 (Yale OpenHand Project-Model T, 2019) which is presented in Section. 2.1.1, **(B)** is the New Dexterity Ultra-Fast Robot Hand (New Dexterity Aerial Grasping Gripper, 2019) which is presented in Section. 2.1.3, **(C)** is an Actively Adaptive Gripper (Kruse and Bradley, 2018) which is presented in Section. 2.1.4, **(D)** is a Permanent Magnet Gripper (Fiaz et al., 2018) which is presented in Section. 2.1.5, **(E)** is the OpenBionics Modular Robotic Gripper (Zisimatos et al., 2014) which is presented in Section. 2.1.2, and **(F)** is a Compliant Bistable Gripper (Zhang H. et al., 2019) which is presented in Section. 2.1.6.

The GRAB Lab at Yale University is one of the leading research groups developing different types of robotic grippers and hands. It is also one of the first groups that developed robotic grippers that can be mounted on aerial platforms. In 2013, Ma et al. developed an adaptive, underactuated robotic gripper which is called the Yale OpenHand Model T, and published it on an open-source library/repository which is called the Yale OpenHand Project (Yale-OpenHand-Project, 2020). The Yale OpenHand Project is one of the first repositories developed for disseminating a series of open-source robot hand and gripper designs (Ma et al., 2013; Odhner et al., 2013, 2014; Ma and Dollar, 2014; Ma et al., 2016; Spiers et al., 2018). The Model T robotic gripper is bio-inspired, and it works similarly to the bird’s claw (Yale OpenHand Project-Model T, 2019). The prototype of this robot gripper has 8 DoF and an adjustable number of actuators when using different types of fingers (fully-actuated or underactuated). When using four underactuated fingers, the fingers with compliant flexure joints are driven by one actuator through a pulley differential mechanism (Ma et al., 2013). The Model T robotic gripper can perform cylindrical, spherical, and planar grasping tasks. It weights 0.49 kg with a holding force up to 13 N, when employing all four underactuated fingers. The Model T gripper is relatively low-cost as it is made by PLA material and rubber, and the users can replace the fingers easily and rapidly as the design is modular. To test the performance of the gripper, it was mounted at the bottom of a mini helicopter and it was used to grasp and carry cylindrical and cubic objects with weights up to around 1.5 kg (Pounds and Dollar, 2010; Pounds et al., 2011; Pounds and Dollar, 2011, 2014). The mechanical structure and appearance of this aerial gripper are demonstrated in Figure 3A. Furthermore, the impact of different design parameters on the performance of aerial grippers, including but not limited to the palm size of the gripper and the link lengths of the fingers and others, were investigated. Based on this investigation, a set of optimal design parameters for aerial grippers were derived and this information can be used to develop aerial grippers with suitable characteristics (Backus et al., 2014; Backus and Dollar, 2017, 2018). This research concluded that underactuated robot grippers and hands with a single motor per finger are sufficient for all the situations involved in aerial applications. However, fully actuated robot grippers and hands equipped with a dedicated motor at each joint can increase perching capabilities of the system.

#### 2.1.2 Control Systems Lab, National Technical University of Athens

To increase the modularity and portability of aerial grippers, researchers of the Control Systems Laboratory at the National Technical University of Athens developed a modular, compliant, underactuated robotic gripper in 2014 and published it on an open-source repository which is called the OpenBionics initiative (OpenBionics, 2020). The prototype of the robotic gripper designed for aerial grasping has 4 DoF and one actuator (a micro servo). The micro servo works together with a differential mechanism, which allows multiple fingers to flex independently being controlled by the same motor. Specifically, the mechanism is capable of controlling all the fingers simultaneously even when one or multiple fingers have stopped moving due to contact with the object surface or other workspace constraints (Zisimatos et al., 2014). Based on this design, the robotic gripper can perform cylindrical, spherical, and planar grasping tasks. It weighs 0.04 kg with a maximum force exertion on each robotic finger up to 18 N. To test the performance of this gripper, it was mounted at the front end of a quadcopter and used to grasp a plastic cup that was loaded with weights. Due to the differential mechanism and the compliance of the robotic gripper, the plastic cup was grasped firmly without significant deformation (OpenBionics-Gripper-UAV-Test, 2020). The mechanical structure and appearance of this aerial gripper is demonstrated in Figure 3E.

#### 2.1.3 New Dexterity Research Group, University of Auckland

In order to perform ultra-fast grasping of fast-moving objects in the air, the New Dexterity research group at the University of Auckland developed an adaptive, underactuated, passive-closing robot hand back in 2017 (Github Repository Aerial Grasping Gripper, 2019; McLaren et al., 2019; New Dexterity Aerial Grasping Gripper, 2019). The prototype of the robot hand has 3 fingers, 6 DoF, and 2 actuators. One of the actuators is used to open the passive closing fingers, and the other motor is used to trigger a quick-release mechanism so that the gripper can be closed instantaneously, facilitating ultra-fast aerial grasping. This robot hand can perform cylindrical, spherical, and planar grasping tasks. It weighs 0.505 kg and it is extremely fast in grasping a plethora of everyday life objects (grasping takes less than 0.1 s). This robot hand is designed for package delivery and the holding force is up to 56 N. To test the performance of this gripper, researchers from the New Dexterity research group mounted it at the bottom of a quadcopter platform and used it to grasp a plethora of everyday life objects. Furthermore, they also used the UAV-gripper system to perform perching tasks on an aluminum pole (New Dexterity Aerial Grasping Gripper, 2019). The mechanical structure and appearance of this aerial gripper are demonstrated in Figure 3B.

#### 2.1.4 NIMBUS Lab, University of Nebraska–Lincoln

To improve the dexterity of manipulation tasks executed with aerial grippers, the NIMBUS Lab of the University of Nebraska–Lincoln developed a hybrid, actively adaptive gripper in 2018 (Kruse and Bradley, 2018). This gripper has 6 DoF and 4 actuators (they work together to grasp target objects with different sizes and shapes from different directions). Furthermore, the robotic gripper utilizes an end-effector design that is curved near the middle to facilitate grasping of cylinders, flat on the edges for grasping cubes, and hollow on each palm for grasping spheres. Based on this design, the gripper can perform cylindrical, spherical, and planar grasping tasks. It weighs 0.297 kg, and it has a holding force that exceeds 0.57 N. To test the performance of this gripper, researchers from the University of Nebraska-Lincoln mounted it at the bottom of a quadcopter platform and used it to grasp a sphere from the top. The mechanical structure and the appearance of this aerial gripper are demonstrated in Figure 3C.

#### 2.1.5 Autonomy Robotics and Cognition Lab, University of Maryland

To improve the payload capacity and stability of aerial grippers, the Autonomy Robotics and Cognition Lab of the University of Maryland cooperated with the King Abdullah University of Science and Technology to develop a permanent magnet robot hand in 2018 (Fiaz et al., 2018). This robot hand has 2 actuators that are employed to create a dual impulsive release mechanism. This gripper can only grasp magnetic objects such as a magnetic box. During the grasping process, the magnetic box is connected with the robot hand utilizing the magnetic force between them. On the other hand, the dual impulsive release mechanism separates them when necessary. Based on this design, thE robot hand can only perform planar grasping tasks involving magnetic objects. It weighs 0.295 kg, and it has a holding force up to 25.48 N. To test the performance of this gripper, it was mounted at the bottom of a quadcopter platform, and it was used to grasp various magnetic objects with planar surfaces. While grasping the objects, the UAV-gripper system successfully performed 90° roll and pitch maneuvers to demonstrate the system’s stability. The mechanical structure and appearance of this aerial gripper is demonstrated in Figure 3D.

#### 2.1.6 Jianguo Zhao’s Lab, Colorado State University

In 2019, Jianguo Zhao’s Laboratory at Colorado State University developed a super light-weight, compliant bistable gripper for mini flying vehicles (Zhang H. et al., 2019). The prototype of this gripper has 3 DoF and 1 actuator used to re-open the bent fingers of the gripper after grasping something. To grasp an object, the gripper is directly activated by the impact force on the palm, and then the state of the gripper switches from open to closed. The structure of the grasping mechanism of the bistable gripper is based on a Von Mises truss, which utilizes the buckling behavior of a truss to change stable states as stated in (Mises, 1923). This gripper can perform cylindrical, spherical, and planar grasping tasks. It weighs 9 g, and it has a holding force up to 0.6 N. To test the performance of the gripper, it was mounted at the top of a mini quadcopter platform, and it was used to perch on a pole as described in (Zhang H. et al., 2019). The mechanical structure and the appearance of this aerial gripper are demonstrated in Figure 3F.

#### 2.1.7 Comparisons and Discussion

**FIGURE 4:**
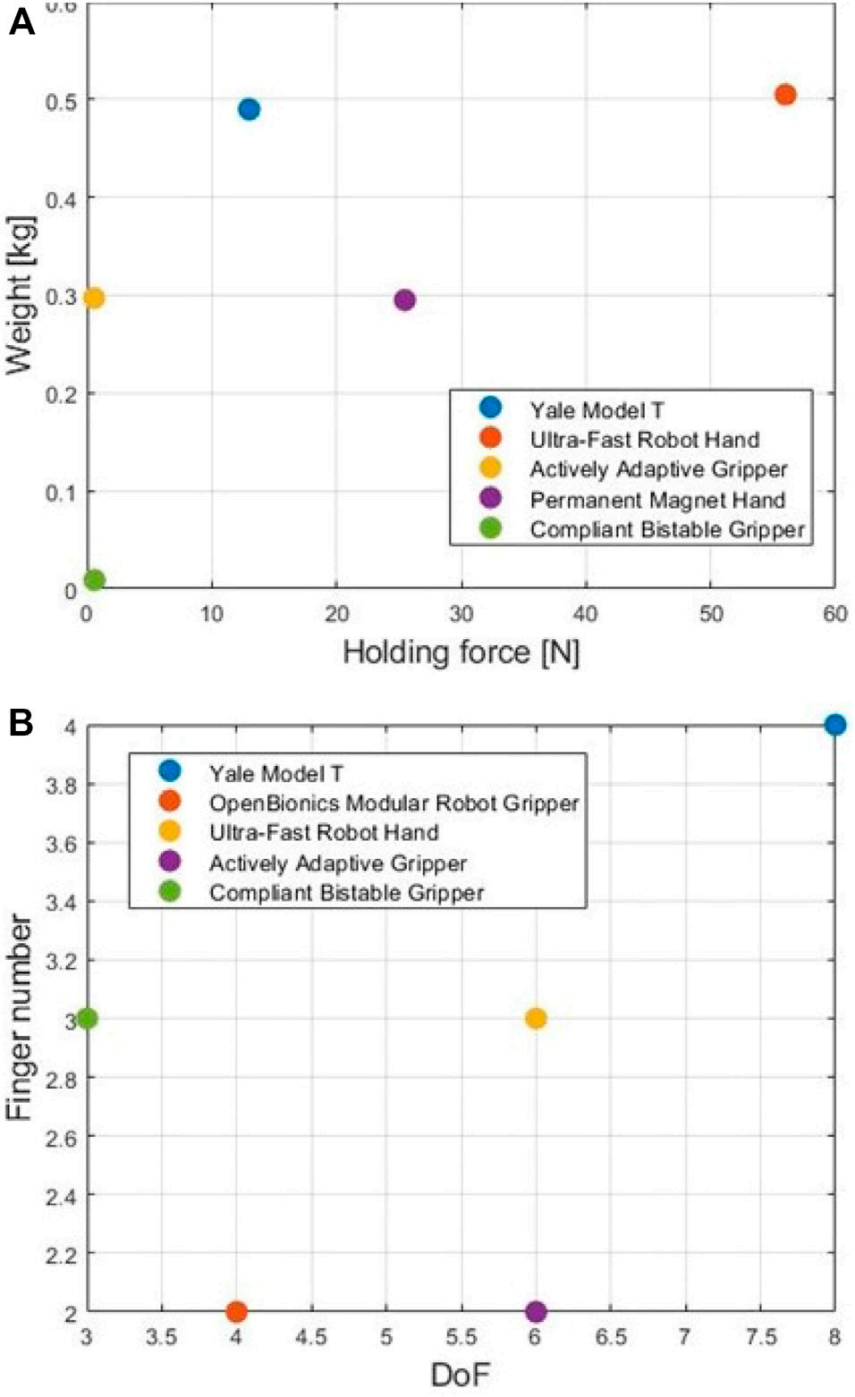
A comparison of the payload capacity and dexterity of the reviewed aerial grippers. Subfigure **(A)** shows a comparison of the weight and holding force of the examined aerial robotic grippers, while **(B)** demonstrates a comparison of the number of fingers and DoF.

In this subsection, we compare and discuss the designs presented in the previous subsections. Figure 3 present the aerial grippers that have been examined. Figure 4A compares their payload capacity by calculating the ratio of the corresponding holding force and the weight of the device. Figure 4B compares the dexterity of the examined devices by calculating the ratio of the corresponding DoF divided by the number of fingers of each device. Finally, Table 2 compares the performances of the grippers as well as characteristics such as the underactuation, the adaptability, and the creation year. Most of the examined aerial grippers are underactuated and adaptive with the only exception being the Permanent Magnet Hand. Although underactuation and adaptability decrease the dexterity of a gripper, they improve grasp stability and decrease the device’s weight and cost. Even though the Permanent Magnet Hand is designed to carry heavy payloads, its payload capacity is still lower than the payload capability of an adaptive aerial gripper such as the Ultra-Fast Robot Hand of the New Dexterity research group (McLaren et al., 2019). Furthermore, aerial grippers with fixed joints such as the Compliant Bistable Gripper have relatively reduced grasping efficiency than aerial grippers with flexible joints.

**TABLE 2:** Comparison of different characteristics of the examined aerial grippers.

Gripper	DoF	Finger	Weight (kg]	Holding force (N]	Underactuated?	Adaptive?	Year
Yale Model T	8	4	0.49	13	Y	Y	2013
OpenBionics Modular Robotic Gripper	4	2	0.04	-	Y	Y	2014
Ultra-fast Robot Hand	6	3	0.505	56	Y	Y	2017
Actively Adaptive Gripper	6	2	0.297	0.57	Y	Y	2018
Permanent Magnet Hand	-	-	0.295	25.48	N	N	2018
Compliant Bistable Gripper	3	3	0.009	0.6	Y	Y	2019

### 2.2 Aerial Manipulation Mechanisms

Long before the advent of commercial multicopters, helicopters were the standard choice for creating grasping capable aerial platforms for the following reasons: 1) the flight of helicopters is relatively stable, and 2) helicopters can hover above a target object and then slowly approach it to grasp it.

#### 2.2.1 GRAB Lab, Yale University

**FIGURE 5:**
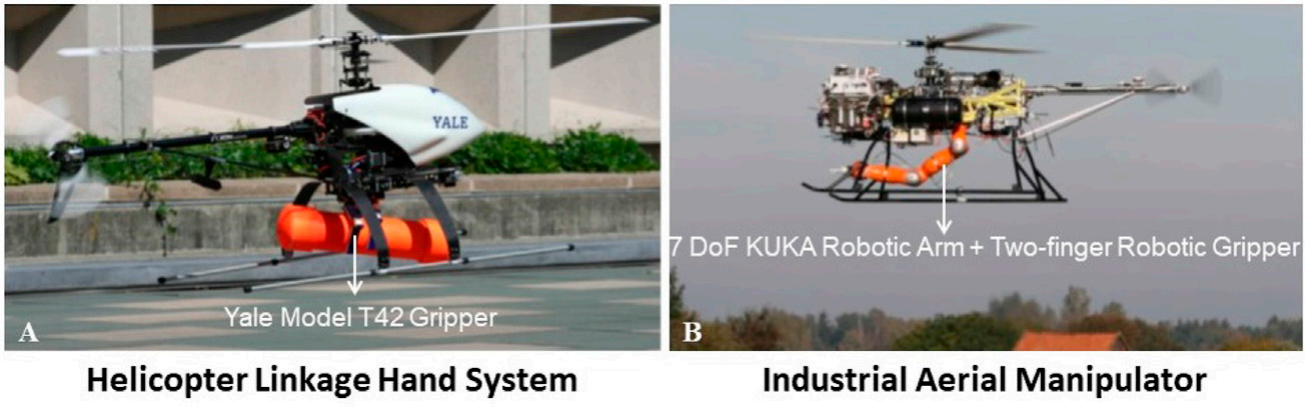
Helicopter-based aerial manipulators for aerial grasping: Subfigure **(A)** presents the Helicopter Linkage Hand System (HLHS) developed by Yale GRAB Lab (Pounds and Dollar, 2010) which is presented in Section. 2.2.1 while **(B)** is the Industrial Aerial Manipulator developed by ARCAS project (Kondak et al., 2014) which is presented in Section. 2.2.2.

In 2010, the GRAB Lab of Yale University developed a Helicopter-linkage-hand System (HLHS) consisting of a helicopter and an aerial gripper 2.1.1 (Pounds and Dollar, 2010; Pounds and Dollar, 2011; Pounds et al., 2011; Pounds et al., 2012). To the best of our knowledge, this is one of the first attempts to develop helicopter-based aerial manipulators. The GRAB Lab researchers proved the stability of the HLHS system by using the Routh-Hurwitz criterion and evaluated its robustness by demonstrating the HLHS would reject dynamic load disturbances. To test the performance of this aerial manipulator, they used the HLHS to grasp and carry cylindrical and cubic objects with weights up to around 1.5 kg (Pounds and Dollar, 2010; Pounds et al., 2011; Pounds and Dollar, 2011, 2014). The mechanical structure and the appearance of this aerial manipulator are demonstrated in Figure 5A.

#### 2.2.2 Institute of Robotics and Mechatronics, German Aerospace Center (ARCAS Project)

In 2014, the Aerial Robotics Cooperatives Assemble System (ARCAS) project of the German Aerospace Center developed an industrial aerial manipulator to carry heavy objects (ARCAS Project, 2019). To accomplish this, a 7 DoF industrial robotic arm was mounted at the bottom of a helicopter to construct an industrial aerial manipulator. As far as we know, this is the first attempt to combine a helicopter and an industrial robotic arm. To test the performance of this industrial aerial manipulator, researchers of the ARCAS project used this industrial aerial manipulator to grasp and carry heavy objects with weights up to around 10 kg (Kondak et al., 2014). The mechanical structure and the appearance of this industrial aerial manipulator are shown in Figure 5B. Furthermore, the ARCAS researchers discovered an unbounded energy flow between the helicopter and the robotic arm in nearly all the situations and that an internal force generated it. A novel kinematic coupling method was proposed to solve this problem, which works by adding an additional DoF of manipulation (in this case, a rotation of the helicopter around the yaw axis) (Kondak et al., 2014).

#### 2.2.3 Institute for Dynamic Systems and Control, ETH Zurich

**FIGURE 6:**
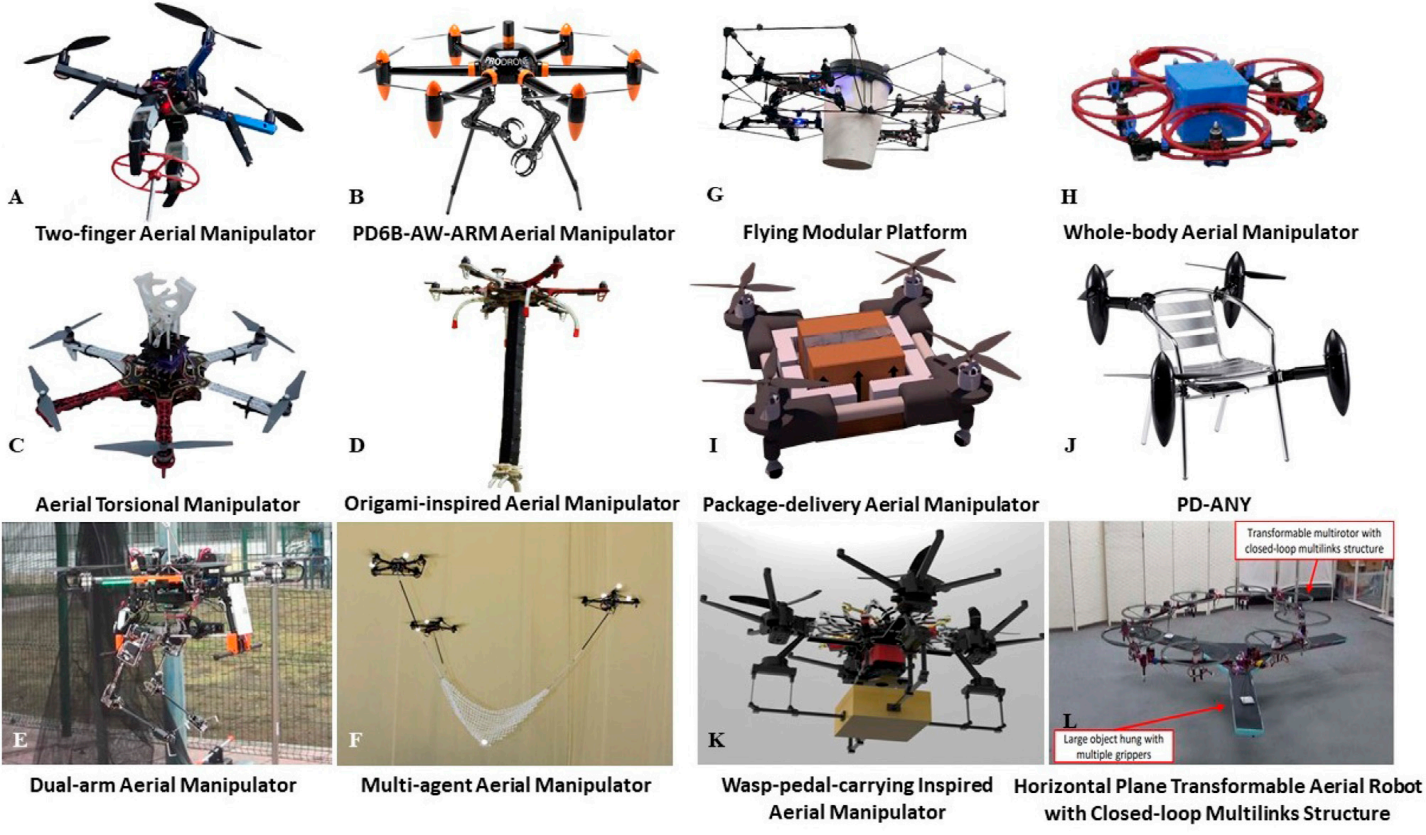
**(A)** to **(F)** shows multicopter-based aerial manipulators for aerial grasping: Subfigure **(A)** presents a Two-finger Aerial Manipulator developed by Drexel University Autonomous System Lab (Orsag et al., 2014) which is presented in Section. 2.2.5, **(B)** is the PD6B-AW-ARM Aerial Manipulator developed by Prodrone company (Prodrone PD6B-AW-ARM from Prodrone Company, 2019) which is presented in Section. 2.2.10, **(C)** is a Aerial Torsional Manipulator developed by Ritsumeikan University (Shimahara et al., 2016) which is presented in Section. 2.2.7, **(D)** is a Origami-inspired Aerial Manipulator developed by Seoul National University Biorobotics Lab (Kim et al., 2018) which is presented in Section. 2.2.9, **(E)** is a Dual-arm Aerial Manipulator developed by University of Seville (Suarez et al., 2017) which is presented in Section. 2.2.8 and **(F)** is a Tether-based, Multi-agent Aerial Manipulator developed by ETH Zurich (Ritz et al., 2012) which is presented in Section. 2.2.3; **(G)** to **(L)** shows reconfigurable frames for aerial grasping: **(G)** is a Flying Modular Platform developed by University of Pennsylvania GRASP Lab (Gabrich et al., 2018) which is presented in Section. 2.3.2, **(H)** is a Whole-body Aerial Manipulator developed by Tokyo University Jouhou System Kougaku Lab (Zhao et al., 2017) which is presented in Section. 2.3.1, **(I)** is a Package-delivery Aerial Manipulator developed by University of Auckland New Dexterity research group (Reconfigurable Drone from New Dexterity Research Team, 2019) which is presented in Section. 2.3.4, **(J)** is the PD-ANY developed by Prodrone company (Prodrone PD-ANY from Prodrone Company, 2019) which is presented in Section. 2.3.5, **(K)** is a Wasp-pedal-carrying Inspired Aerial Manipulator developed by University of Nevada and Beijing Institute of Technology which is presented in Section. 2.3.3, and **(L)** is a Horizontal Plane Transformable Aerial Robot with Closed-loop Multilinks Structure (HALO) developed by Tokyo University Jouhou System Kougaku Lab (Anzai et al., 2018) which is presented in Section. 2.3.1.

To increase the dexterity of aerial manipulation, the Institute for Dynamic Systems and Control at ETH Zurich developed a tether-based, multi-agent aerial manipulation system (Ritz et al., 2012). The multi-agent system consists of four quadcopters and a lightweight net (weighs only 120 g). Each of these quadcopters is connected with one corner of the net. Based on an accurate formation control algorithm, the multi-agent system can dynamically catch or throw an object in the air. As far as we know, this is one of the first attempts to develop multi-agent aerial manipulators. The performance of this aerial manipulation solution was tested by using it to catch a ping-pong ball and then throw it in the air several times, achieving a very high success rate (Ritz et al., 2012). The mechanical structure and the appearance of this aerial manipulation system are demonstrated in Figure 6F.

#### 2.2.4 GRASP Lab, University of Pennsylvania

To increase the efficiency of aerial manipulation systems, the GRASP Laboratory at the University of Pennsylvania developed an avian-inspired aerial manipulator in 2013 (Thomas et al., 2013, 2014; Mohta et al., 2014). This aerial manipulator consists of a quadcopter, a robotic arm, and a two-finger robotic gripper, and its control algorithm imitates the predatory behavior of avian species. More specifically, it can perform perching at relatively high ground and then quickly glide down to grasp the target object on the ground. To the best of our knowledge, this is the first attempt to imitate the grasping behavior of avian feet. To ensure the stability of grasping, the gripper has two wide fingers that can surround the objects maximizing the contact area. Thus, the stability of the grasps that are performed with this aerial manipulator can be guaranteed by design. Detailed information about the gripper such as the mechanical structure and the working principle have been presented in (Thomas et al., 2013). To demonstrate the similarity of this avian-inspired aerial manipulator and a real eagle, the researchers performed a comprehensive comparison in (Thomas et al., 2014). They also investigated and proposed a perching algorithm for this aerial manipulator in (Mohta et al., 2014). Although the particular avian-inspired aerial manipulator was published in 2014, it is still one of the most successful bio-inspired aerial manipulators.

#### 2.2.5 Autonomous Systems Lab, Drexel University

In order to make UAVs capable of navigating hard-to-access locations such as narrow spaces inside a factory and perform dexterous tasks, the Autonomous Systems Laboratory at Drexel University developed a small two-finger aerial manipulator in 2014 (Orsag et al., 2014; Korpela et al., 2014). More specifically, two fully actuated robotic fingers were mounted at the bottom of a mini quadcopter to construct this aerial manipulator. The small volume of the aerial manipulator makes it capable of accessing narrow spaces to perform various dexterous tasks such as detecting cracks on the wall or open/close valves. To test the performance of this aerial manipulator, it was used for opening/closing hydraulic valves many times, achieving a very high success rate (Orsag et al., 2014; Korpela et al., 2014). The mechanical structure and the appearance of this aerial manipulator are demonstrated in Figure 6A.

#### 2.2.6 Autonomous Systems Lab, ETH Zurich

To increase the stability of an UAV system during the process of carrying payloads, the Autonomous Systems Laboratory of ETH Zurich developed a lightweight aerial manipulator consisting of a mini quadcopter and a folding robotic arm back in 2015 (Bellicoso et al., 2015). More specifically, the robotic arm can fold, constraining the CoG of the entire system during the process of carrying payloads and during landing. Hence the total inertia and static unbalance of the whole system can be significantly reduced. The maximum extension, weight, and payload of this robotic arm are 300 mm, 250 and 200 g, respectively.

#### 2.2.7 Integrated Sensor and Intelligence Lab, Ritsumeikan University

To better conduct daily tasks that require the exertion of torsional forces, such as replacing lamp bulbs and bolts on the ceiling of buildings and harvesting fruits, Ritsumeikan University developed an aerial torsional manipulator back in 2016 (Shimahara et al., 2016). This aerial torsional manipulator consists of a quadcopter and a gripper. The gripper is mounted at the top of the quadcopter, making it possible for this aerial torsional manipulator to grasp objects from the bottom. The width, height, and weight of the aerial torsional manipulator are 790 mm, 400 mm, and 1910 g, respectively. To test the performance of this aerial manipulator, it was used to unscrew a light bulb that required a torque of 4 Nm. (Shimahara et al., 2016) The mechanical structure and the appearance of this aerial manipulator are demonstrated in Figure 6C.

#### 2.2.8 Robotics, Vision, and Control Group, University of Seville

In 2017, the University of Seville developed a lightweight (weights approximately 1.3 kg), dual-arm aerial manipulator with an aluminum structure designed for carrying the robotic arms (Suarez et al., 2017; Caballero et al., 2018; Suarez et al., 2018b,a). The aluminum structure can protect the actuators against direct impacts and overloads during the grasping process. Specifically, the slightly compliant frame can absorb the energy of impacts and overloads passively at higher rates, allowing active energy release at lower rates (Suarez et al., 2017). To test the performance of the aerial manipulator, it was used to grasp objects with weights up to 0.6 kg. Furthermore, they discovered that the ability of the compliant joints to resist impacts and joint overloads is associated with the motion constraints. By considering appropriate motion constraints, the success rate of the aerial manipulator in various grasping tasks can be significantly improved as stated in (Caballero et al., 2018; Suarez et al., 2018b,a). The mechanical structure and the appearance of this aerial manipulator are demonstrated in Figure 6E.

#### 2.2.9 Biorobotics Lab, Seoul National University

In 2018, the Biorobotics Laboratory of the Seoul National University developed an origami-inspired aerial manipulator (Kim et al., 2018). It consists of a hexacopter and an origami-inspired robotic arm that can fold to be flat. The origami principle of perpendicular folding enables the shape and stiffness of the robotic arm to be changed by a single actuator. Furthermore, this robotic arm can achieve an extension-to-compression ratio of 17.5:1, enabling the origami-inspired manipulator to grasp objects in hard-to-access locations such as underwater environments. To test the performance of this origami-inspired manipulator, it was used to grasp objects in various outdoor environments, such an object in a ditch, an object on a tree branch, or an object located underwater (Kim et al., 2018). The mechanical structure and the appearance of this aerial manipulator are demonstrated in Figure 6D.

#### 2.2.10 Prodrone Company

The Prodrone company has also developed a dual-arm aerial manipulator (PD6B-AW-ARM) for commercial applications. PD6B-AW-ARM consists of two slim robotic arms and a hexacopter platform (Prodrone PD6B-AW-ARM from Prodrone Company, 2019). This can be regarded as a bio-inspired design because the arms of the aerial manipulator are aesthetically and functionally similar to the legs of a swan. Based on the official information provided in (Prodrone PD6B-AW-ARM from Prodrone Company, 2019), PD6B-AW-ARM is waterproof, and the weight, flight duration, maximum flight speed, and maximum payload capacity of the platform are 19 kg, 15 min, 60 km/h, and 20 kg, respectively. The mechanical structure and the appearance of this aerial manipulator are demonstrated in Figure 6B.

#### 2.2.11 Comparisons and Discussion

In Figure 5 and Figure 6, we present the aforementioned aerial manipulators’ mechanisms. Table 3 presents a comparison of the characteristics of the examined aerial manipulators, the stability, and the production year. In Table 3, most of the aerial platforms presented are quadcopters as the stability of a quadcopter platform is adequate in most cases. There are still some aerial manipulators that employ hexacopters as aerial platforms, such as the Origami-inspired Aerial Manipulator because its grasping process has a higher requirement on overall stability. However, the use of a hexacopter platform increases the overall size and weight of the aerial manipulator.

**TABLE 3:** Comparison of characteristics of the examined aerial manipulators for aerial grasping.

Aerial manipulator	Aerial platform	Specific type	Interaction process	Specific type	*P*_*g*_[*%*]	Stability?	Year
HLHS	Helicopter	-	Gripper	-	3.5	Y*	2010
Multi-agent Aerial Manipulator	Multicopter	Multi-agent system with 4 quadcopters	Gripper			Y*	2012
Avian-inspired Aerial Manipulator	Multicopter	Quadcopter	Arm-gripper	Single-arm	24	N	2013
Industrial Aerial Manipulator	Helicopter	-	Arm-gripper	Single-arm	12.5	Y*	2014
Two-finger Aerial Manipulator	Multicopter	Quadcopter	Arm-gripper	Dual-arm	-	Y*	2014
Folding-arm Aerial Manipulator	Multicopter	Quadcopter	Arm-gripper	Folding-arm	13.2	Y	2015
Aerial Torsional Manipulator	Multicopter	Hexacopter	Gripper	-	17.5	Y	2016
Dual-arm Aerial Manipulator	Multicopter	Quadcopter	Arm-gripper	Dual-arm	23	Y*	2017
Whole-body Aerial Manipulator	Reconfigurable frame	Revolute joint	Reconfigurable frame	Revolute joint	100	Y	2017
HALO	Reconfigurable frame	Revolute joint	Reconfigurable frame	Revolute joint	100	Y*	2017
Origami-inspired Aerial Manipulator	Multicopter	Hexacopter	Arm-gripper	Folding-arm	20.6	Y	2018
Wasp-pedal-carrying Aerial Manipulator	Multicopter	Quadcopter	Reconfigurable frame	Revolute joint	4	Y	2018
Flying Modular Platform	Reconfigurable frame	Revolute joint	Reconfigurable frame	Revolute joint	100	Y	2018
Package-delivery Aerial Manipulator	Reconfigurable frame	Prismatic joint	Reconfigurable frame	Prismatic joint	100	Y	2018
PD-ANY	Reconfigurable frame	Revolute joint	Reconfigurable frame	Revolute joint	100	Y	-
PD6B-AW-ARM	Multicopter	Hexacopter	Arm-gripper	Dual-arm	-	N	-

**Notes:** $P_{g}=\frac{w_{i}}{w_{t}}$ , where *P*_*g*_ is the proportion of the interaction tool of each aerial manipulator for aerial grasping, *w*_*i*_ is the weight of the interaction tool, end-effector and *w*_*t*_ is the total weight of the aerial manipulator. The stability of aerial manipulators depends on whether the COGs of the target object and the aerial manipulator are optimized: i) Y means that stability is achieved by a mechanism, ii) Y* means that stability is achieved by an algorithm, and iii) N means that neither the mechanism nor the control algorithm of the aerial manipulator would help improve stability.

### 2.3 Reconfigurable Drones for Aerial Grasping

Drones with reconfigurable frames are a new class of aerial manipulators that can be used for autonomous aerial grasping and transportation. They can be viewed as transformable, shape-shifting multicopters, which can grasp and transport the target objects by altering the shape of their frame. Compared with multicopters, the reconfigurable drones have the following benefits: 1) they can provide a highly stable grasping process utilizing the large contact area between the reconfigurable frame and the object, and 2) they can provide a highly stable transportation process as the reconfigurable frame allows for an alignment of the CoGs of the object and the frame.

#### 2.3.1 Jouhou System Kougaku Lab, Tokyo University

In 2017, the Jouhou System Kougaku Laboratory of Tokyo University developed a novel transformable aerial manipulator which is called Horizontal Plane Transformable Aerial Robot with Closed-loop Multilinks Structure to pick up large objects (Zhao et al., 2016; Anzai et al., 2017, 2018). This platform can transform into different shapes in the horizontal plane based on the closed-loop, multi-link structure with gripping units, presented in (Zhao et al., 2016). In order to improve the stability of the aerial manipulator, the researchers proposed a shape adaptive transformation algorithm in (Anzai et al., 2017). This algorithm is designed to align the CoG of the target object with the CoG of platform. Based on the structure of aerial manipulator and the algorithm, the platform can carry large payloads through the cooperation of multiple aerial modules, while avoiding collisions among them. Since this platform can transform itself into a line shape, it becomes more versatile in passing through narrow gaps compared with square-shaped aerial manipulators (Anzai et al., 2018). The mechanical structure and the appearance of this aerial manipulator are demonstrated in Figure 6L. In 2017, the same laboratory proposed a multi-link, whole-body aerial manipulator based on the linkage structure presented in (Zhao et al., 2017; Zhao et al., 2018 M.). Compared with the previous platform, this whole-body aerial manipulator has no additional gripper attached to each aerial module. There are three main stages for this whole-body aerial manipulator to grasp an object: firstly, it transforms into the right shape, secondly, it approaches and surrounds the object, thirdly, it supports the object relying on the friction between the object surface and the aerial manipulator’s surface. To measure the stability and robustness of the aerial manipulator, its adaptation for various objects such as cubic objects and cylindrical objects was tested and the torque and grasping angles during the transportation process, were measured in (Zhao et al., 2017; Zhao et al., 2018 M.). The mechanical structure and the appearance of this aerial manipulator, are demonstrated in Figure 6H.

#### 2.3.2 GRASP Lab, University of Pennsylvania

To increase the adaptability of reconfigurable frames and better protect the propellers, the GRASP Laboratory of the University of Pennsylvania developed a flying modular platform that can carry objects with relatively small sizes (Gabrich et al., 2018). The module has a cuboid frame with a locking mechanism. Different number of modules can form various linkage systems that surround target objects, cage them, and then transport them. To test the performance of this aerial manipulator, it was used to grasp a coffee cup and transport it to a trash can (Gabrich et al., 2018). The mechanical structure and the appearance of this aerial manipulator are demonstrated in Figure 6G.

#### 2.3.3 Yantao Shen’s Research Group, University of Nevada

In 2018, Yantao Shen’s research group at the University of Nevada cooperated with the Beijing Institute of Technology to develop a wasp-pedal-carrying inspired, deformable aerial manipulator for aerial grasping and transportation (Zhao N. et al., 2018). The weight of the aerial manipulator is about 1 kg and its payload capacity is 0.3 kg. This aerial manipulator is capable of deforming due to the proposed rigid elements based morphing structure, which is able to control the grasping mechanism so as to expand or contract. This mechanical structure exhibits a good performance of balancing the CoGs of the aerial manipulator and the target objects as the target object is always at the center of the rigid elements based morphing structure. To test the performance of the wasp-pedal-carrying inspired aerial manipulator, it was used to grasp everyday life objects such as a package box, a baseball and cans of different sizes (Zhao N. et al., 2018). The mechanical structure and the appearance of this aerial manipulator are demonstrated in Figure 6K.

#### 2.3.4 New Dexterity Research Group, University of Auckland

To develop reconfigurable drone frames for the application of aerial package delivery, the New Dexterity research group at University of Auckland developed a reconfigurable structure that can be used for transporting cubic objects such as box-shaped packages (Reconfigurable Drone from New Dexterity Research Team, 2019). This aerial manipulator has an extensible linkage frame with prismatic joints and can easily grasp different sizes of cubic objects such as box-shaped packages as stated in (Reconfigurable Drone from New Dexterity Research Team, 2019). More specifically, the cubic object can be enclosed within the aerial manipulator during the transportation process; therefore, the CoG of the entire system including the aerial manipulator and the cubic object is constant during the transportation process and this significantly improves the stability of the transportation process. The mechanical structure and the appearance of this aerial manipulator are demonstrated in Figure 6I.

In 2020, the same research group at University of Auckland proposed an ultra-lightweight, net-based reconfigurable frame (Hingston et al., 2020). This aerial manipulator utilises twisted string actuation to pull together eight fingertips mounted at the lower net end so as to grasp target objects. The net-based grasping mechanism of the reconfigurable frame is ultra-light-weight (weights around 76 g), and it allows for autonomously loading and unloading payloads. To test the performance of the net-based aerial manipulator, the platform was used to grasp everyday life objects with different sizes and weights such as a banana, a fork, a wooden block, a plastic bottle, a football and the maximum payload capability of this aerial manipulator is 12.08 kg (Hingston et al., 2020).

#### 2.3.5 Prodrone Company

The Prodrone company has also developed a flying module platform named PD-ANY for commercial applications (Prodrone PD-ANY from Prodrone Company, 2019). PD-ANY is a single propeller platform that can be used to build an aerial manipulator together with the payload. More specifically, PD-ANY can turn a payload into a part of the aerial manipulator during the building process. The CoG of the aerial manipulator can be shifted to its center so as to increase stability, so users can select different numbers of PD-ANYs to form an aerial manipulator while placing PD-ANYs at the optimal positions. Based on the official information provided in (Prodrone PD-ANY from Prodrone Company, 2019), PD-ANY is waterproof and the weight, flight duration, and maximum flight speed of the platform are about 4 kg, 10 min, and 20 km/h, respectively. The mechanical structure and the appearance of this aerial manipulator are demonstrated in Figure 6J.

#### 2.3.6 Comparisons and Discussion

From the results of Table 3, it is evident that in recent years researchers tend to use mechanisms to ensure the stability of aerial manipulators rather than algorithms. For example, the emerging reconfigurable frames directly replace the transitory multi-agent systems. This is because using mechanisms has a distinct advantage on the development cycle, efficiency, and overall cost performance. In addition, aerial manipulators with robotic arms increase the dexterity of the grasping and manipulation, but the proportion of the weight of the interaction tool to the weight of the entire system *P*_*g*_, also increases considerably.

## 3 Aerial Perching Mechanisms

In this section, we present and analyze the research conducted on aerial robots with perching mechanisms.

### 3.1 Grasping-Based Perching Mechanisms

Grasping is one of the most intuitive perching technologies. In the nature, many birds perch on tree branches or other terrains by using the grasping capability of their claws. Grippers that have been reviewed in Section. 2.1 with large holding force can also be considered as proper aerial perching mechanisms. Grasping-based perching mechanisms use a simple take-off process, as when the gripper starts getting released the thrust-to-weight ratio of the aerial robot is gradually increasing allowing for take-off.

#### 3.1.1 Robotic Systems Lab, University of Utah

**FIGURE 7:**
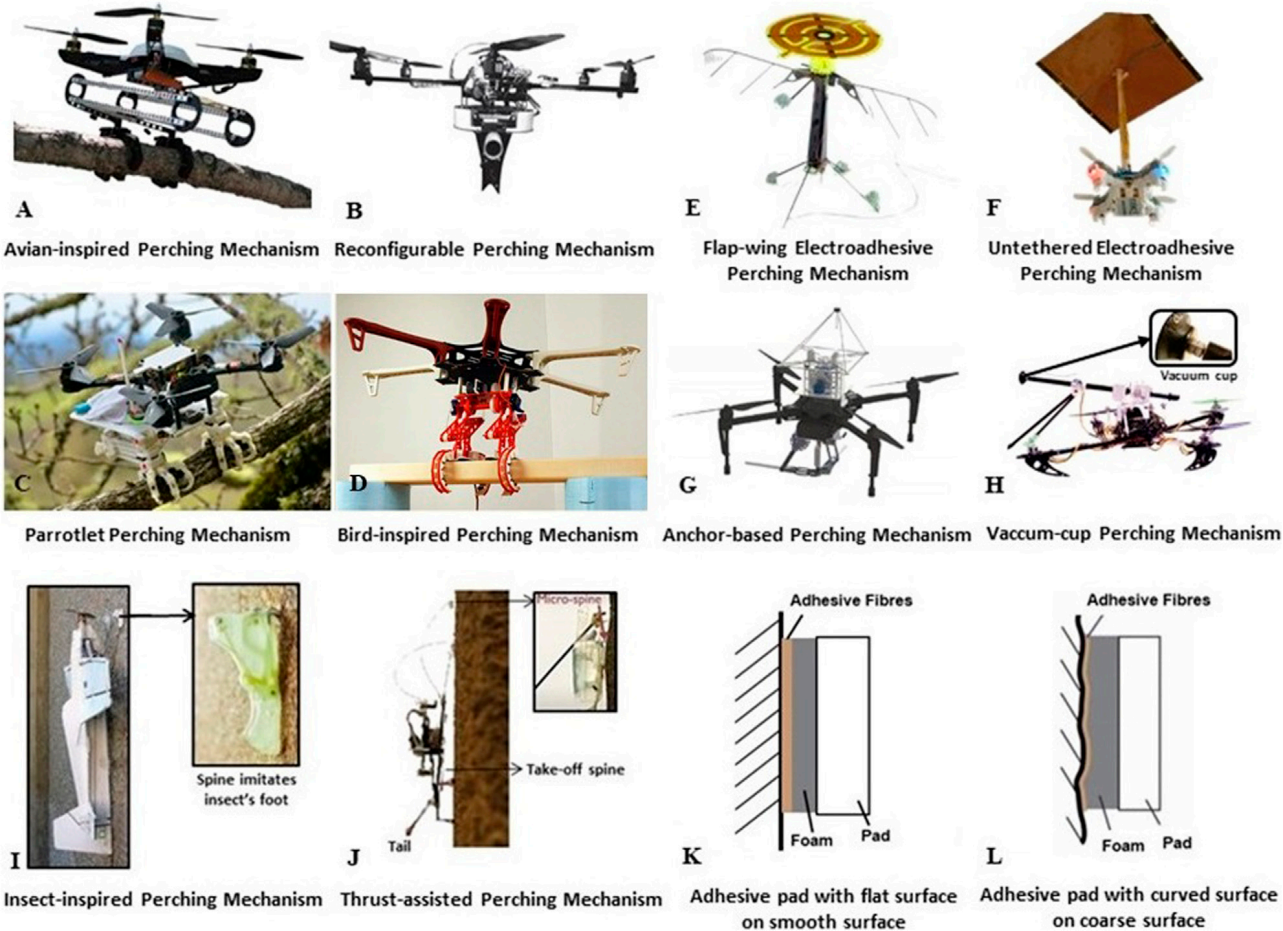
Subfigures **(A)** to **(D)** demonstrate aerial manipulators with grasping mechanisms for aerial perching: **(A)** is an Avian-inspired Perching Mechanism developed by University of Utah Robotic Systems Lab (Doyle et al., 2012) which is presented in Section. 3.1.1, **(B)** is a Reconfigurable Perching Mechanism developed by University of Southampton Autonomous Systems Lab (Erbil et al., 2013) which is presented in Section 3.1.2, **(C)** is a Parrotlet Perching Mechanism developed by Standford University Biomimetics and Dextrous Manipulation Lab (Roderick et al., 2021) which is presented in Section 3.1.6 and **(D)** is a Bird-inspired Perching Mechanism developed by Olin College of Engineering (Nadan et al., 2019) which is presented in Section. 3.1.5; **(E)** to **(H)** demonstrate aerial robots and aerial manipulators with attaching mechanisms for aerial perching: **(E)** is a Flap-wing Electroadhesive Perching Mechanism developed by MIT, Harvard University, City University of Hong Kong, University of Washington and Illinois Institute of Technology (Graule et al., 2016) which is presented in Section. 3.3.3, **(F)** is a Untethered Electroadhesive Perching Mechanism developed by Stanford University Power Electronics Research Lab (Park et al., 2020) which is presented in Section. 3.3.7, **(G)** is an Anchor-based Perching Mechanism developed by Imperial College London Aerial Robot Lab (Zhang et al., 2017) which is presented in Section. 3.3.6 and **(H)** is a Vacuum-cup Perching Mechanism developed by University of Twente Robotics, Vision and Machine Intelligence Lab (Wopereis et al., 2016) which is presented in Section. 3.3.4; **(I)** to **(J)** demonstrate aerial manipulator and aircraft with embedding mechanisms for aerial perching: **(I)** is an Insect-inspired Aerial Perching Mechanism developed by Stanford University Biomimetics and Dextrous Manipulation Lab (Desbiens and Cutkosky, 2010) which is presented in Section. 3.2.1 and **(J)** is a Thrust-assisted Perching Mechanism developed by Stanford University Biomimetics and Dextrous Manipulation Lab (Pope et al., 2016) which is presented in Section. 3.2.1; **(K)** to **(L)** demonstrate a comparison of adhesive pads with different surfaces: **(K)** presents an adhesive pad with flat fibre and foam that has better adhesion performance on smooth surface, and **(L)** presents an adhesive pad with curved fibre and foam that has better adhesion performance on coarse surface.

In 2012, the Robotic Systems Laboratory of the University of Utah developed an avian-inspired, passive perching mechanism for UAVs (Doyle et al., 2012). Its design imitates songbirds perching on tree branches while sleeping. The perching mechanism has two compliant, underactuated claws and two folding legs. This perching mechanism transforms the weight of the UAV into tendon tension and then passively drives the claws. More specifically, the tendons on the back ankle help the claws to grasp the branch tightly when the legs are folded. This perching mechanism weighs only 478 g and the UAV platform weighs 388 g. To test the performance of this perching mechanism, it was used to help a quadcopter perch on different terrains. With this perching mechanism, perching success rates of 98% can be achieved as stated in (Doyle et al., 2012). The mechanical structure and appearance of this mechanism are demonstrated in Figure 7A.

#### 3.1.2 Autonomous Systems Lab, University of Southampton

In 2016, the Autonomous Systems Laboratory of the University of Southampton developed a reconfigurable perching mechanism (weighs 1.5 kg) for UAVs so as to allow them to land on the ground or perch on a rod (Erbil et al., 2013). When the reconfigurable perching mechanism is opened, it can be used to land on the ground. On the other hand, when the reconfigurable perching mechanism is closed, it can be used to perch on a rod. The efficiency of the proposed perching mechanism was experimentally validated with experiments that involved wind speeds of up to 4.6 km/s (Erbil et al., 2013). The mechanical structure and the appearance of this perching mechanism are demonstrated in Figure 7B.

#### 3.1.3 New Dexterity Research Group, University of Auckland

The aerial gripper that has been reviewed in Section. 2.1.3 and proposed in 2017 can also be used to perform reliable aerial perching behaviour (New Dexterity Aerial Grasping Gripper, 2019). To test the aerial perching performance of this gripper, researchers from New Dexterity team mounted it at the bottom of a quadcopter (DJI F450 Flame Wheel Drone) and used the UAV-gripper system to perch on poles (the diameter of these poles are up to 50 mm). In experiments, they discovered this aerial gripper can support a system with a weight up to 5.22 kg in perching tasks (New Dexterity Aerial Grasping Gripper, 2019). The mechanical structure and appearance of this perching mechanism are demonstrated in Figure 7C.

#### 3.1.4 Jianguo Zhao’s Lab, Colorado State University

The aerial gripper that has been reviewed in Section. 2.1.6 and proposed in 2019 can also be used to perform reliable aerial perching for mini flying vehicles (Zhang H. et al., 2019). To test the performance of this gripper, researchers of the Jianguo Zhao’s Laboratory mounted it at the top of a mini quadcopter (Carzyflie 2.0) and used it to help the UAV-gripper system to perch on a pole during experiments. The mechanical structure and the appearance of this perching mechanism can be found in (Zhang H. et al., 2019).

#### 3.1.5 Chris Lee’s Research Group, Olin College of Engineering

In 2019, the Chris Lee’s Research Group at the Olin College of Engineering developed a bird-inspired perching mechanism (Nadan et al., 2019). The working principle of the perching mechanism is inspired by the anatomy of birds that grasp and perch as tendons in their legs and feet are tensioned. Compared with the avian-inspired perching mechanism in Section. 3.1.1, this perching mechanism employed a different mechanical structure on the leg to transform the weight of the UAV into tendon tension. For detailed information about the leg structure please refer to (Nadan et al., 2019). To test the performance of this perching mechanism, it was mounted under different UAV platforms to help them perch on different terrains. The mechanical structure and the appearance of this perching mechanism are demonstrated in Figure 7D.

#### 3.1.6 Biomimetics and Dextrous Manipulation Lab, Stanford University

In 2021, the Biomimetics and Dextrous Manipulation Laboratory at Stanford University developed a parrotlet perching mechanism with a weight of 250 g that allows quadcopters to perching on cylindrical terrains (Roderick et al., 2021). This perching mechanism imitates parrotlet landing and perching behaviours on tree branches in the nature. The perching mechanism contains two important internal mechanisms found in many bird legs to improve grasping performance, namely the digital flexor mechanism (DFM) and the tendon locking mechanism (TLM). We briefly describe DFM as a mechanism that the tendons bend the toes are routed around the ankle. Therefore, the tendons can be tensioned when the leg bends at the ankle. In the TLM, tendons passing through each toe interact with locking features in their associated tendon sheaths. Thus, the toes are locked onto the objective surface when the bird's claw closes actively. A series of outdoor experiments were conducted and the results demonstrated that the designed perching mechanism can transform the impact energy into grasp force in many high-speed collision cases. During the collision process, the perching mechanism can wrap around irregularly shaped objects such as tree branches and hold them firmly within 50 ms. The mechanical structure and the appearance of this perching mechanism are demonstrated in Figure 7C.

### 3.2 Embedding-Based Perching Mechanisms

Embedding-based perching is very common in a plethora of insects in the Nature. This perching approach can be applied to inclined or vertical rough surfaces. Normally, the stability of this perching technology is in-between the stability offered by grasping and attaching.

#### 3.2.1 Biomimetics and Dextrous Manipulation Lab, Stanford University

In 2010, the Biomimetics and Dextrous Manipulation Laboratory at Stanford University developed a insect-inspired perching mechanism with a weight of only 28 g that allows aircraft to perch on vertical walls (Desbiens and Cutkosky, 2010; Lussier Desbiens et al., 2011; Glassman et al., 2012). This perching mechanism imitates the tiny spines on the insects’ foot that can embed into the cracks on the wall or hang on the raised part on the wall. A series of outdoor experiments were conducted in order to test the performance of an aircraft equipped with this perching mechanism at the bottom. The aircraft achieved 30 successful perching trials out of a total of 40 trials with a flight speed of about 3 m/s. Most of the unsuccessful trials were due to the inaccuracy of the employed ultrasonic sensor. When testing the aircraft in indoor experiments without winds or other disturbances, it achieved 110 successful perching trials out of a total of 114 trials and the success rate achieved was 96.5%. Following the steps below, the aircraft that is perched on the vertical wall begins to take off again in three steps: first, the propeller speed is gradually increasing simultaneously with the thrust-to-weight (T/W) ratio after setting the aileron trim and enabling the attitude control; second, the spines are released just before takeoff using the spine release mechanism (it retracts the spines in about 150 ms) and the T/W ratio immediately increases; third, with the increased T/W ratio, the aircraft sprints up for a certain distance and then gradually smooths out (Desbiens and Cutkosky, 2010; Lussier Desbiens et al., 2011; Glassman et al., 2012). The mechanical structure and the appearance of this perching mechanism are demonstrated in Figure 7I.

In 2015, the same laboratory developed a thrust-assisted perching mechanism, which is called the Stanford Climbing and Aerial Maneuvering Platform (Pope and Cutkosky, 2016; Pope et al., 2016). This perching mechanism also imitates the tiny spines on the insects’ foot that can embed into the cracks on the wall or hang on the raised part on the wall. It consists of a tail, two front spines, a take-off spine and a spring-based extend/retract system. There are four main steps for a quadcopter equiped with this perching mechanism to perch, climb, and take-off on the wall: firstly, the quadcopter with this perching mechanism would fly towards the wall until the tail contacts it; secondly, the quadcopter would align the body with the wall using the thrust from rotors with the tail acting as a pivot; thirdly, once the quadcopter aligns with the wall, it can climb the wall using the spring-based extension/retraction system; finally, the take-off spine would be employed so that the quadcopter can release two front spines to take-off. For detailed information about the process please refer to (Pope and Cutkosky, 2016). This perching mechanism weighs only 11 g and it was attached on a quadcopter that weighs 28 g. Based on the experiments conducted, we know that the employed quadcopter needed only 10% of the maximum thrust to hold its position on walls (Pope and Cutkosky, 2016; Pope et al., 2016). The mechanical structure and the appearance of this perching mechanism are demonstrated in Figure 7J.

#### 3.2.2 Aerial Robot Lab, Imperial College London

In 2019, the Aerial Robot Laboratory at Imperial College London developed a passive adaptive microspine perching mechanism with a weight of only 32 g that allows multicopters to perch on different cylindrical terrains such as tree branches and pipelines (Nguyen et al., 2019). This perching mechanism consists of multiple compliant grapple modules. Each module is formed from individual plastic links with a trapezoidal cross-section and uses cable tension to make the module curl. A pair of sharpened steel spines protrude from the underside of each link of the compliant grapple module to increase the perching capability of the device. In addition, these sharpened steel spines can be replaced by magnets. Once the perching mechanism contacts a cylindrical object such as a tree branch, it warps around the tree branch firmly and tension is applied. The perching mechanism can be released easily when the tension is released by gradually increasing the T/W ratio of the multicopter. To test the performance of this perching mechanism, it was used on a quadcopter weighing about 1.7 kg to perch on a variety of cylindrical objects such as tree branches (ash, oak, and balsa) and steel pipes. This mechanism demonstrates a high load capability with a maximum tension of up to 60 kg in some cases (Nguyen et al., 2019).

### 3.3 Attaching-Based Perching Mechanisms

Perching technology based on the attachment process became very popular during recent years as it can be applied on both coarse and smooth surfaces. A smooth surface is the most difficult terrain in all the perching scenarios. Currently, existing perching technologies based on the attachment process include 1) magnets, 2) dry adhesives, 3) electrostatic adhesives, and 4) vacuum cups.

#### 3.3.1 Intelligent Systems Lab, EPFL

In 2013, the Intelligent Systems Laboratory of EPFL developed a fiber-based adhesive perching mechanism to allow multicopters to perch on vertical surfaces such as walls no matter if they are coarse or smooth (Daler et al., 2013). This perching mechanism contains several compliant deployable pads and a passive self-alignment system to increase the contacting area of the deployable pads. When using this perching mechanism, active controllers are not required. To test the performance of this perching mechanism, it was mounted on a light-weight quadcopter (weighs about 0.3 kg) and it was used to help the quadcopter perch on a wall. This perching mechanism allows the quadcopter to perch on a wall by simply employing the mechanism and flying towards the wall. In order to take off again, the quadcopter must increase the T/W ratio and retract the perching mechanism (Daler et al., 2013). In experiments, the researchers further discovered that fiber-based adhesives with curved surfaces have better performance on coarses wall than fiber-based adhesives on flat surface, as the former can sink into the small grooves of the wall. However, fiber-based adhesives with flat surfaces have better performance on smooth surfaces than fiber-based adhesive on curved surfaces as the former create larger contact areas with the wall. A detailed demonstration of this discovery can be found in Figure 7K,L.

#### 3.3.2 Robotics Lab, Illinois Institute of Technology

In 2015, the Robotics Laboratory of the Illinois Institute of Technology developed a three-directional dry adhesive based perching mechanism for UAVs (Kalantari et al., 2015). In this design, the perching mechanism comprises three directional dry adhesive pads arranged in a triangular configuration. The mechanism’s attachment and detachment are performed employing a single servo motor. By simply flying towards and touching the target surfaces, this perching mechanism allows UAVs to exhibit robust perching behaviors. To test the performance, the mechanism was used on a micro UAV. The total weight of the system is around 550 g. Furthermore, researchers incorporated force sensors in each pad to detect the loading condition and let the system move towards the target surface at different speeds. Based on the experiments conducted, it was shown that the perching mechanism requires a UAV moving speed of more than 0.42 m/s to provide an impact force of more than 4 N to achieve successful perching. Once the moving speed of the UAV is more than 0.4 m/s, the success rate of the experimental trials is more than 93% (Kalantari et al., 2015).

#### 3.3.3 Microrobotics Lab, Harvard University

In 2016, research groups in Harvard University, MIT, City University of Hong Kong, University of Washington, and Illinois Institute of Technology collaborated to develop a flap-wing electroadhesive perching mechanism, which can perch on horizontal surfaces from the bottom side (Chirarattananon et al., 2014a,b,c; Chirarattananon and Wood, 2013; Chirarattananon et al., 2016; Graule et al., 2016). More specifically, they designed an insect-inspired aerial robot with two flapping wings at the middle, four tripods at the bottom, and an electroadhesive patch at the top. The electroadhesive patch weighs about 13.4 mg and is 15% of the total mass of the aerial robot; meanwhile, it can provide 15.6 Pa pressure for the aerial robot to perch on coarse and smooth surfaces when operating at 1000 V voltage. Furthermore, this perching mechanism can be used in environments with humidity up to 70% as the electrodes are built inside the electroadhesive patch. There are two steps that allow the aerial robot to perch on an object and then take off: firstly, when contacting the object, the electrodes generate surface charge between the object’s surface and the electroadhesive patch to provide an attraction force; secondly, when the power supply for the electrodes is switched off, the attraction force disappears and the aerial robot can fly away. To test the performance of this perching mechanism, it was used on aerial robots to perch on various materials such as wood, glass, and plant (Chirarattananon et al., 2014a,b,c; Chirarattananon and Wood, 2013; Chirarattananon et al., 2016; Graule et al., 2016). The mechanical structure and the appearance of this perching mechanism are demonstrated in Figure 7E.

#### 3.3.4 Vision and Machine Intelligence Lab, University of Twente

In 2016, the Robotics, Vision and Machine Intelligence Laboratory at the University of Twente developed a vacuum-cup perching mechanism that weighs approximately 0.3 kg (Wopereis et al., 2016). This perching mechanism consists of a spring-based, passive buffer device with a vacuum cup mounted at the front and two tripods. This perching mechanism uses direct contact to perch on the vertical surface and uses a wire to pull the vacuum cup to detach. This perching mechanism allows aerial manipulators to perform reliable and reversible perching behaviours on vertical smooth surfaces such as walls. To test the performance of this perching mechanism, it was used to assist a quadcopter with a weight of 1.8 kg to perform stable perching on a wall. The quadcopter was also able to detach the vacuum cup and take-off. The mechanical structure and the appearance of this perching mechanism are demonstrated in Figure 7H.

#### 3.3.5 GRASP Lab, University of Pennsylvania

In 2016, the GRASP Laboratory at the University of Pennsylvania developed a gecko-inspired, dry adhesive perching mechanism with a weight of 60 g (Thomas et al., 2016). This perching mechanism is influenced by the opposed-grip dry adhesive design described in (Hawkes et al., 2016) and consists of four silicone rubber pads that can provide a relatively large friction even when attaching to smooth surfaces. Furthermore, this perching mechanism is light-weight as the silicone rubber pads can be pulled or released simultaneously employing a single motor. As soon as the perching mechanism contacts the surface, the load tendon is pulled with a truss collapse mechanism and engaged with adhesive to support the load. When the load is removed by increasing the T/W ratio, the adhesive passively disengages and the perching mechanism can easily disengage from the surface. This perching mechanism can ensure reliable perching behaviors on inclined or even vertical surfaces. To test the performance of the mechanism, it was used to help quadcopters with different weights to perch on inclined smooth surfaces with an adhesive pressures of up to 10 kPa (Thomas et al., 2016).

#### 3.3.6 Aerial Robot Lab, Imperial College London

In 2017, the Aerial Robot Laboratory of the Imperial College London developed a spider-inspired, anchor-based perching mechanism (Zhang et al., 2017; Braithwaite et al., 2018). This perching mechanism allows UAVs to perform reliable perching behaviours in specific indoor environments. This perching mechanism consists of four anchor launchers and a string spooling system that tensions the anchors. Furthermore, each anchor has a magnetic ring mounted at the front and a string connected at the end. To test the performance of this perching mechanism, it was used to help quadcopters to perform perching in indoor environments with iron doors. When the quadcopter decides to perch, the anchors are launched and are attached on the iron doors and then the string spooling system is employed to tension the strings (Zhang et al., 2017; Braithwaite et al., 2018). The mechanical structure and the appearance of this perching mechanism are demonstrated in Figure 7G.

#### 3.3.7 Power Electronics Research Lab, Stanford University

In 2020, the Power Electronics Research Laboratory at Stanford University developed an untethered electroadhesive perching mechanism (Park et al., 2020). This perching mechanism allows mini UAVs to perch on horizontal surfaces such as the ceiling. More specifically, the power supply is mounted at the bottom of the mini UAV and the mini UAV is connected with the electroadhesive pad through a cable at the top. The mini UAV’s perching and takeoff processes are similar to the flap-wing electroadhesive perching mechanism described in 3.3.3. To test the performance of this perching mechanism, it was used to help quadcopters of different weights (up to 1,300 g) to perch on the ceiling with different power supply voltages (from 0.5 V to 4.3 KV). Furthermore, the mechanism allows the aerial vehicle to remain perched on a horizontal surface, no matter if it is coarse or smooth, for about 100 min, which is almost 15 times longer than the aerial vehicle’s flight time (Park et al., 2020). The mechanical structure and the appearance of the perching mechanism are demonstrated in Figure 7F.

#### 3.3.8 Mechanical System and Vibration Lab, Shanghai Jiao Tong University

In 2020, the Mechanical System and Vibration Laboratory at Shanghai Jiao Tong University developed a dual elasticity combined suction cup (DEC-cup) to assist multicopters to perch on vertical walls under disturbances (Liu et al., 2020). The DEC-cup consists of an inner soft cup and an outer firm cup that facilitate the execution of the perching process without reducing the adhesion stiffness. When suction cups are used to perform perching, in many cases, improper contact angle or insufficient contact force can result to failures. In this design, the inner soft cup is adaptable to the angular error caused by the multicopter allowing for adaptation to the angular error between the outer firm cup and the contact surface and secure perching on a surface. Hence perching can well be done with this mechanism without requiring precise control to ensure success. To test the performance of the DEC-cup, it was used on a quadcopter weighing 1.77 kg to perch on a vertical wall. The quadcopter perches on the vertical wall and then takes off again in two steps: 1) the quadcopter autonomously opens the vacuum pump and releases the valve, while flying towards and touching the wall; 2) the quadcopter gradually increases the T/W ratio while closing the vacuum pump to take off (Liu et al., 2020).

#### 3.3.9 Zhifeng Huang’s Lab, Guangdong University of Technology

In 2021, the Zhifeng Huang’s Laboratory at Guangdong University of Technology developed a magnetic extended-leg perching mechanism that can enable fast-moving UAVs to perch on inclined or vertical surfaces without requiring speed reduction before touchdown (Huang et al., 2021). This perching mechanism has a weight of 10 g and consists of four extended legs with small magnetic pads mounted at the front ends. The mechanism can be attached to various aerial platforms such as quadcopters. This perching mechanism utilizes a flip-and-flap perching strategy. More specifically, once an aerial platform with this perching mechanism touches the target surface, the kinetic energy of the aerial platform is converted into potential energy by flipping upward with the extended legs. After the aerial platform reaches the maximum altitude, it performs a pendulum-like motion following a downward swing and a PD controller is triggered to generate thrust while dissipating previous generated potential energy, allowing the aerial system to perch on the surface smoothly. To test the performance of this perching mechanism, it was used on a Crazyflie 2.0 quadcopter that has a total weight of 32 g and it was used to perch on vertical surfaces at different speeds. A maximum flying speed of around 2.8 m/s, ensured a high success rate for perching (Huang et al., 2021).

### 3.4 Comparisons and Discussion

Figure 7 presents the aforementioned aerial perching mechanisms and Table 4 presents a comparison of the characteristics of the examined aerial perching mechanisms such as the applicable perching terrains and the proportion of the weight of the perching mechanism to the entire weight of each aerial platform used for interacting. As demonstrated in Table 4, the majority of the aerial platforms are multicopters as they were the most widely used during the past decade and the perching of multicopters is easier than the perching of flap-wing robots and aircrafts. Grasping-based perching mechanisms are widely used to attach to cylindrical objects, embedding-based perching mechanisms are widely used to attach to planar terrains with coarse surfaces, and attaching-based perching mechanisms are widely used to attach to planar terrains with smooth and coarse surfaces. Also, grasping-based perching mechanisms are heavier than the other perching mechanisms, in most cases, as presented in Table 4.

**TABLE 4:** Comparison of characteristics of the examined aerial manipulators for aerial perching.

Perching mechanism	Aerial platform	Specific type	Interaction process	Specific type	Perching on planar objects?	Perching on cylindrical objects?	*P*_*p*_[*%*]	Year
Insect-inspired Perching Mechanism	Mini aircraft	-	Embedding	-	Y	N	10	2010
Avian-inspired Perching Mechanism	Multicopter	Quadcopter	Grasping	-	N	Y	55	2012
Fibre-based Perching Mechanism	Multicopter	Quadcopter	Attaching	Dry adhesive	Y	N	-	2013
Thrust-assisted Perching Mechanism	Multicopter	Quadcopter	Embedding	-	Y	N	28	2015
Three-directional Perching Mechanism	Multicopter	Quadcopter	Attaching	Dry adhesive	Y	N	-	2015
Flap-wing Electroadhesive Perching Mechanism	Flap-wing robot	-	Attaching	Electrostatic adhesive	Y	N	15	2016
Reconfigurable Perching Mechanism	Multicopter	Hexarotor	Grasping	-	N	Y	22	2016
Vacuum-cup Perching Mechanism	Multicopter	Quadcopter	Attaching	Vacuum cup	Y	N	17.5	2016
Gecko-inspired Perching Mechanism	Multicopter	Quadcopter	Attaching	Dry adhesive	Y	N	10.3	2016
Anchor-based Perching Mechanism	Multicopter	Quadcopter	Attaching	Magnet	Y	N	-	2017
Bird-inspired Perching Mechanism	Multicopter	Quadcopter	Grasping	-	N	Y	-	2019
Adaptive Microspine Perching Mechanism	Multicopter	Quadcopter	Embedding	-	N	Y	1.9	2019
Untethered Electroadhesive Perching Mechanism	Multicopter	Quadcopter	Attaching	Electrostatic adhesive	Y	N	9.8	2020
DEC-cup Perhing Mechanism	Multicopter	Quadcopter	Attaching	Vacuum cup	Y	N	-	2020
Magnetic Extended-leg Perching Mechanism	Multicopter	Quadcopter	Attaching	Magnet	Y	N	31.25	2021
Parrotlet Perching Mechanism	Multicopter	Quadcopter	Grasping	-	N	Y	33.3	2021

**Notes:** $P_{p}=\frac{w_{p}}{w_{t}}$ , where *P*_*p*_ is the proportion of the perching mechanism of each aerial manipulator used for interacting, *w*_*p*_ is the weight of the perching mechanism and *w*_*t*_ is the total weight of the aerial manipulator. Planar objects include inclined and vertical surfaces such as walls and cylindrical objects include poles such as tree branches.

## 4 Discussion and Analysis

In this section, we discuss the findings of the work presented in the previous sections and then we analyze the advantages and the disadvantages of the corresponding technologies.

### 4.1 Aerial Grasping Mechanisms

The grippers that are suitable for aerial grasping and that we reviewed in this paper have, in most cases, two or three fingers. These configurations are adequate for grasping objects through underactuated designs without requiring the execution of complex in-hand manipulation motions. Regarding the payload capacity of the grippers, all of them have the ability to lift an object that has at least the same weight as the gripper. Based on our observations, underactuated grippers are characterised by significant payload capacities due to the use of fewer actuators. This is due to the fact that the weight of additional actuators on fully-actuated grippers increases the weight of the device and fully-actuated grippers are more prone to failures due to the high dependence on static torque. Moreover, some aerial grippers (such as the Ultra-fast gripper presented in Section. 2.1.3 and the Compliant bistable gripper presented in Section. 2.1.6) also employed different types of quick-release mechanisms that trigger the grasping process passively. Quick-release mechanisms can help aerial grippers to grasp fast-moving objects in the air. Comparing fully-actuated grippers such as the Active Adaptive Gripper presented in 2.1.4 with the underactuated grippers that are equipped with quick-release mechanisms, such as the Ultra-fast Robot Hand presented in 2.1.3, the former have more advantages in within-hand manipulation capability and the latter have more advantages in grasping speed and payload capacity. In addition, the grippers with a quick-release mechanism should employ motors with higher force exertion capabilities, as the quick-release mechanism requires a relatively large static torque to be pretensioned.

**FIGURE 8:**
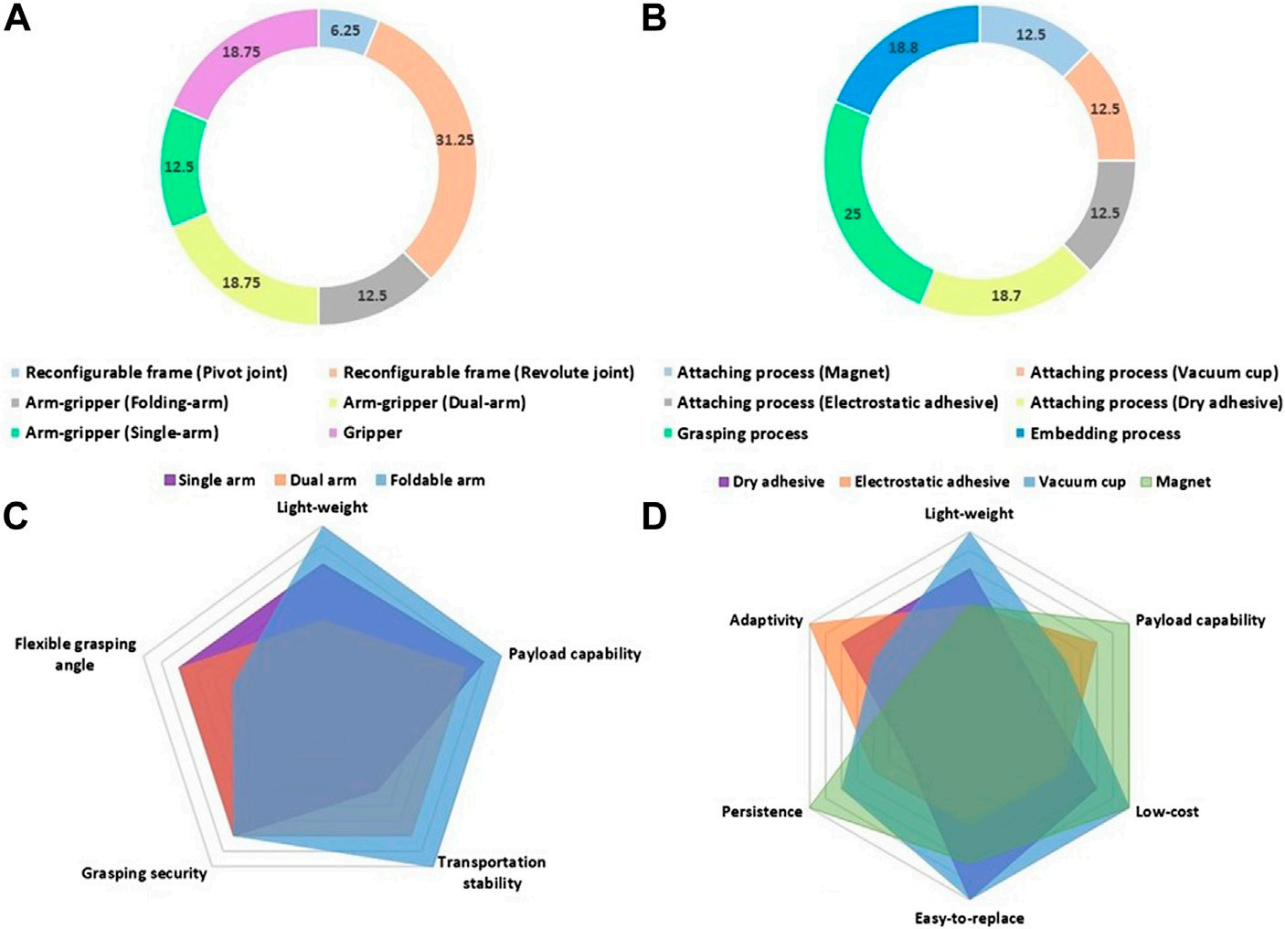
A proportion analysis of the mentioned aerial robots with grasping and perching capabilities is demonstrated in the first two sub-figures: Subfigure **(A)** presents the proportion analysis of mentioned aerial robots with grasping capabilities and **(B)** presents the proportion analysis of mentioned aerial robots with perching capabilities; A comparison of the mentioned mainstream grasping (arm-gripper) and perching (attaching) technologies is demonstrated in the last two sub-figures: **(C)** presents the comparison of different arm-gripper technologies across five aspects including weight, payload capability, transportation stability, grasping security, and flexibility of grasping angle and **(D)** presents the comparison of different attaching technologies across six aspects including weight, payload capability, cost, replaceability, persistence, and adaptivity.

For the aerial robots with grasping capabilities that we have reviewed in this paper, the aerial platforms employed include helicopters, multicopters, and reconfigurable drones, and their interaction platforms include grippers, arm-gripper systems, reconfigurable frames and multi-agent systems. The proportion analysis of these aerial robots with grasping capabilities is presented in Figure 8A, the comparison of different aerial grasping technologies is presented in Table 5, and the comparison of different arm-gripper technologies is presented in Figure 8C. For the aerial robots with grasping capabilities that we have reviewed, most of the aerial platforms are quadcopters and hexacopters, as their flexibility is adequate to support most of the aerial grasping tasks and applications. Although the helicopter was the dominant aerial platform in the early years of aerial vehicles due to its high stability, it has been gradually replaced by multicopters as it lacks flexibility in many cases such as when navigation of narrow passages is needed. As mentioned earlier, mounting a gripper on an aerial platform seems to be the most intuitive way to achieve aerial grasping. However, compared with the arm-gripper system, it lacks in dexterity and manipulation capability due to the limited grasping and manipulation workspace. In addition, disturbance from the propellers can also be a problem for the gripper system, as its grasping distance is relatively short compared with the arm-gripper system. Adding a robotic arm between the gripper and the aerial platform is a good solution for overcoming the aforementioned problems. However, the arm-gripper system (single-arm) may also lead to other problems, increasing swing or instability of the robotic arm during the transportation process. To solve this issue, some researchers have proposed to use dual robotic arms to enhance stability and others have proposed to use a folding robotic arm. Compared with employing a folding robotic arm, employing dual robotic arms can not fully align the CoGs of the target object and the aerial platform during the transportation process. Meanwhile, aerial robots with dual robotic arms tend to be heavier than aerial robots with folding robotic arms. As a result, we believe that folding a robotic arm is a better option compared to the use of two robotic arms. Reconfigurable frames also are an excellent solution for aerial grasping due to the following reasons: 1) they can perfectly align the CoGs of the target object and the aerial platform by integrating the target object as a part of the frame, 2) they are relatively light-weight as they don’t need robotic arms or grippers, and 3) they can grasp target objects with relatively large sizes as they are not limited by the length of the robotic fingers. More specifically, reconfigurable frames with revolute joints seem to have better adaptability on objects with different shapes and sizes (see Figure 6H). On the other hand, reconfigurable frames with prismatic joints can provide robust grasping for objects with a specific shapes (see Figure 6I). Finally, multi-agent systems require complex control and path planning algorithms for determining the motion of each aerial unit and appropriate formation algorithms for coordinating all the aerial units simultaneously, which leads to an increased complexity in algorithmic design.

**TABLE 5:** Advantages and disadvantages of the discussed aerial grasping and perching technologies.

Grasping Technologies	Advantages	Disadvantages
Gripper	1. Light-weight	1. Grasping angle is limited
	2. High stability when moving objects	2. Cannot grasp large objects
Arm-gripper (Single-arm)	1. Grasping angle is not limited	1. Lack stability when moving objects
	2. High security when grasping objects	2. Cannot grasp large objects
Arm-gripper (Dual-arm)	1. Grasping angle is not limited	1. Heavy
	2. Relatively high stability when moving objects	2. Cannot grasp large objects
	3. High security when grasping objects	
Arm-gripper (Folding-arm)	1. Relatively light-weight	1. Grasping angle might be limited
	2. High stability when moving objects	
	3. High security when grasping objects	
Reconfigurable frame (Revolute joint)	1.High stability when moving objects	1. Grasping angle is limited
	2. Can grasp large objects with various shapes	
Reconfigurable frame (Prismatic joint)	1. Very high stability when moving objects	1. Grasping angle is limited
	2. Can grasp large objects with one specific shape	2. Cannot grasp objects with various shapes
Multi-agent system	1. Can grasp large objects with various shapes	1. Hard to control when moving objects
	2. Grasping angle is not limited	2. Relatively heavy
**Perching Technologies**	**Advantages**	**Disadvantages**
Embedding	1. Light-weight	1. Cannot work on smooth surface
	2. High stability	2. Cannot perform cylindrical perching behavior
	3. Good payload capacity	
	4. Good persistence	
	5. Good performance on planar perching behavior	
Grasping	1. Very high stability	1. Relatively heavy
	2. Good payload capacity	2. Cannot perform planar perching behaviors
	3. Good persistence	
	4. Good performance on cylindrical perching behavior	
Attaching (Dry adhesive)	1. Light-weight	1. Poor payload capacity
	2. Low cost	2. Lack persistence
	3. Easy-to-replace	3. Cannot perform cylindrical perching behavior
	4. Can work on smooth surface	
Attaching (Electrostatic adhesive)	1. Light-weight	1. Hard-to-produce
	2. Can work on smooth surface	2. Require extra power supply
		3. Cannot perform cylindrical perching behavior
Attaching (Vacuum cup)	1. Light-weight	1. Cannot perform cylindrical perching behavior
	2. Low cost	2. Cannot work on coarse surface due to gas leak
	3. Easy-to-replace	
	4. Can work on smooth surface	
Attaching (Magnet)	1. High stability	1. Cannot work on non-magnetic objects
	2. Good payload capacity	2. Cannot perform cylindrical perching behavior
	3. Good persistence	3. Hard to detach from the objects

### 4.2 Aerial Perching Mechanisms

For the reviewed aerial robots with perching capabilities, their aerial platforms include bio-inspired robots, multicopters, and fixed-wing aircrafts, and their interaction processes include attaching, grasping, and embedding. The proportion analysis of these aerial robots with perching capabilities, is presented in Figure 8B, the comparison of different aerial perching technologies, is presented in Table 5, and the comparison of the different attaching technologies, is presented in Figure 8D. Aerial perching is a newly-developed field and the majority of the developed aerial perching mechanisms are inspired by the behaviours of animals such as various birds and insects. For the aerial robots with perching capabilities that we have reviewed in this paper, most of their aerial platforms are quadcopters and hexacopters, as their flexibility is adequate to support most of the aerial perching tasks and applications. Perching mechanisms based on grasping approaches seem to be the most intuitive and robust to use on cylindrical structures, such as tree branches. Grippers used for perching on cylindrical structures require the ability to lift an object that has at least the same weight as the entire UAV system (the gripper and the aerial platform). Perching on coarse or even smooth surfaces is more difficult than perching on cylindrical structures. Perching mechanisms based on the embedding process work well on coarse surfaces as they can hang on the raised parts or leaks of coarse surfaces. However, they cannot work on smooth surfaces as there are no raised parts or leaks for the embedding mechanism to hang. Perching mechanisms based on the attaching process can be categorized to four main categories, including: 1) dry adhesives, 2) vacuum cups, 3) electrostatic adhesives, and 4) magnets. Dry adhesives can work on both coarse and smooth surfaces, but they lack robustness in many cases, as the stickiness decreases over time. Vacuum cups can only work on smooth surfaces as the raised parts or leaks on the coarse surfaces lead to air leaks. Both the performances of dry adhesives and vacuum cups are affected by the humidity of the environment. Nevertheless, electrostatic adhesives work well on both coarse and smooth surfaces no matter the influence of the humidity of the environment. The only drawback of electrostatic adhesives is the requirement of an extra power supply, which adds extra weight on the aerial robot. Finally, magnets can only work under specific conditions and their attaching force is powerful, but the detaching process is problematic.

## 5 Conclusion

In this paper, we reviewed, presented, and compared several aerial robots with grasping and perching capabilities that have been developed over the past decade. We first reviewed grippers that are designed for aerial grasping and also aerial robots with specific mechanisms (e.g., reconfigurable frames) that facilitate aerial grasping. Underactuated grippers with quick-release mechanisms demonstrated a distinct advantage in grasping fast-moving objects and carrying heavy payloads for a relatively long duration. Quick-release mechanisms can transfer the stress from actuators to other mechanical structures on the gripper. For instance, the reviewed ultra-fast robot hand that was presented in Section 2.1.3 can transfer the stress from the actuator of the tendon-driven system (when it is not triggered) to the passive-closing, soft robotic fingers (when it is triggered). A fully-actuated gripper with a multifunctional end-effector can perform various sophisticated manipulations like the Actively Adaptive Gripper presented in Section 2.1.4 that has been used to pour a beer. In terms of aerial robots with manipulation capabilities, manipulators with a folding robotic arm and reconfigurable frames have distinct advantages compared to other mechanisms and approaches. More specifically, a manipulator with a folding robotic arm can grasp objects from different angles at a relatively distant location, hence minimising the disturbance from the rotating propellers and it can fold during the transportation process, minimising the swing of the robotic arm. Reconfigurable drones can conform to the object shape and securely grasp objects of various sizes and geometries, increase the contact area between the object and manipulator and maximising stability during the transportation process. But due to the change of the position of the propellers, the shape-shifting process of reconfigurable frames incurs a higher control complexity. In general, the control algorithm for a reconfigurable frame would not be similar to others as there is a significant difference in the overall structure of a reconfigurable drone. This might also be the reason why reconfigurable drones require longer design and development cycles. In the second part of the review, we reviewed, presented, and compared aerial robots with perching capabilities. More specifically, grasping-based aerial perching mechanisms attach well to cylindrical objects/terrains by holding them firmly. The minimum holding requirement for a grasping-based aerial perching mechanism is to hold the total weight of the perching mechanism and the aerial platform. Furthermore, embedding-based aerial perching mechanisms attach well to rough inclined or vertical surfaces as they can hang on the raised parts or leaks of the rough surfaces. Attaching-based aerial perching mechanisms attach well to both rough and smooth surfaces as they can maximise the contact area between the perching mechanism and the surfaces. Finally, we analyzed the advantages and the disadvantages of the corresponding technologies (Table 5) and we discussed the major accomplishments in these fields (Table 6).

**TABLE 6:** Major achievements of laboratories, research groups, and companies for aerial robots with grasping and perching mechanisms.

Institution	Major achievements
Yale University	1. Developed a series of fully-actuated and underactuated aerial grippers with high replaceability and portability based on tendon-driven mechanisms.
GRAB Lab	
(www.eng.yale.edu/grablab/)	
	2. Built an open-source library / repository (Yale OpenHand Project) to benefit researchers across the world in the field of robotic hands.
	3. Systematically researched control algorithms for Helicopter-Linkage-Hand System (HLHS).
	4. Systematically researched the difference between the perching and resting behaviour of an aerial robot.
National Technical University of Athens	1. Developed a modular, compliant, underactuated aerial gripper with high replaceability and portability based on tendon-driven mechanisms.
Gontrol Systems Lab	
(www.controlsystemslab.gr/main/)	
	2. Built an open-source library / repository (OpenBionics) to benefit researchers across the world in the field of developing robotic and bionic devices.
University of Auckland	1. Developed an adaptive, underactuated, ultra-fast aerial gripper based on a tendon-driven, quick-release mechanism for grasping fast-moving objects in the air and perching on cylindrical objects.
New Dexterity research team	
(newdexterity.org/)	
	2. Developed a series of reconfigurable drones for aerial grasping and package delivery.
University of Nebraska–Lincoln	1. Developed a hybrid, actively adaptive gripper with an end-effector design that is curved near the middle to facilitate grasping of cylinders, flat on the edges for grasping cubes, and hollow on each palm for grasping spheres.
NIMBUS Lab	
(justinbradley.unl.edu/about)	
University of Maryland	1. Developed a permanent magnet robot hand with dual impulsive release mechanism to grasp magnetic objects such as a magnetic box.
Autonomy Robotics Cognition Lab	
(robotics.umd.edu/facilities/autonomy-robotics-cognition-lab)	
Colorado State University	1. Developed a super light-weight, compliant bistable gripper based on Von Mises Truss for mini flying vehicles to perch on cylindrical objects.
Jianguo Zhao’s Lab	
(www.engr.colostate.edu/∼zhao/)	
German Aerospace Center	1. Developed an industrial aerial manipulator that consists of a helicopter and a 7 DoF industrial robotic arm to grasp and carry heavy objects.
Institute of Robotics and Mechatronics	
(www.dlr.de/rm/en/)	
ETH Zurich	1. Systematically researched control algorithms of the cooperative grasping behaviour of multi-agent UAVs.
Institute for Dynamic Systems and Control	
(idsc.ethz.ch/)	
ETH Zurich Autonomous Systems Lab (asl.ethz.ch/)	1. Developed an aerial manipulator with a folding robotic arm to constrain the CoG during grasping.
University of Pennsylvania	1. Systematically researched control algorithms of an avian-inspired aerial manipulator including gliding down to grasp objects and perching on cylindrical objects.
GRASP Lab	2. Developed a mini reconfigurable frame based on a linkage locking mechanism that can grasp objects with various sizes and shapes.
(www.grasp.upenn.edu/)	
	3. Developed a gecko-inspired, dry adhesive perching mechanism for aerial manipulators to perch on inclined smooth surfaces.
Drexel University	1. Developed a super light-weight aerial manipulator with two fully-actuated fingers mounted at its bottom, which can perform dexterous grasping behaviours and manipulation tasks such as turning valves.
Autonomous Systems Lab	
Ritsumeikan University	1. Developed an aerial torsional manipulator that can grasp objects from the bottom and then rotate itself. For example, it can screw / unscrew a light bulb.
Integrated Sensor and Intelligence Lab	
(www.ritsumei.ac.jp/se/∼skazu/index_e.html)	
University of Seville	1. Developed a light-weight, dual-arm aerial manipulator with an aluminum frame structure designed for the robotic arms. The aluminum frame structure can protect the actuators against direct impacts and overloads during the grasping process.
Robotics, Vision and Control Group	
(grvc.us.es/)	
Seoul National University	1. Developed an aerial manipulator with an origami-inspired, self-locking, foldable robotic arm to grasp objects in hard-to-access locations and constrain the CoG during grasping.
Biorobotics Lab	
(www.biorobotics.snu.ac.kr/)	
Prodrone Company	1. Developed a goose-inspired, dual-arm, commercial-grade aerial manipulator that can carry heavy payloads with a high moving speed.
(www.prodrone.com/ )	
	2. Developed an easy-to-replace, portable, commercial-grade reconfigurable frame that can turn any payload into a part of it before taking off.
Tokyo UniversityJouhou System Kougaku Lab	3. Developed a series of adaptive, multi-link reconfigurable frames that can carry objects with different shapes.
(www.jsk.t.u-tokyo.ac.jp/)	2. Systematically researched control algorithms for reconfigurable frames, which can constrain the CoGs of reconfigurable frames when grasping and carrying objects.
EPFL	1. Developed a fiber-based, adhesive perching mechanism that can enable aerial robots to perch on smooth vertical surfaces based on the attaching process.
Intelligent Systems Lab	
(www.epfl.ch/labs/lis/)	
Harvard University	1. Developed a flap-wing electroadhesive perching mechanism that can perch on horizontal surfaces from the bottom side.
Microrobotics Lab	
(www.micro.seas.harvard.edu/)	
University of Twente	1. Developed a passive, vacuum-cup perching mechanism that can enable aerial robots to perch on smooth vertical surfaces based on the attaching process.
Robotics, Vision and Machine Intelligent Lab	
Imperial College London	1. Developed a magnetic, anchoring mechanism that can enable aerial robots to perch on smooth, magnetic surfaces based on the attaching process.
Aerial Robot Lab	
(www.imperial.ac.uk/aerial-robotics)	
	2. Developed a passive adaptive microspine perching mechanism that allows multicopters to perch on different cylindrical terrains such as tree branches and pipelines.
Stanford University	1. Developed an untethered, electroadhesive perching mechanism that can enable mini aerial robots to perch on smooth surfaces based on the attaching process.
Power Electronics Research Lab	
(superlab.stanford.edu/)	
Stanford University	1. Developed an insect-inspired perching mechanism that can enable aerial robots to perch on walls based on the embedding process.2. Developed a perching mechanism that can enable aerial robots to perform robust grasping-based perching behaviour on cylindrical terrains.
Biomimetics and Dextrous Manipulation Lab	
(bdml.stanford.edu/)	
University of Uath	1. Developed an avian-inspired, passive perching mechanism that imitates the behaviour of a bird’s legs when perching on a tree branch. This mechanism can enable aerial robots to perch on cylindrical objects based on the grasping process.
Robotic Systems Lab	
(my.mech.utah.edu/∼minor/index.html)	
University of Southampton	1. Developed a reconfigurable perching mechanism that can enable aerial robots to perch on cylindrical objects and to land on the ground.
Autonomous Systems Lab	
Olin College of Engineering	1. Developed a bird-inspired perching mechanism with a differential mechanism on the leg of the perching mechanism to utilize the weight of the UAV to increase tendon tension.
Chris Lee’s Research Group	
(http://faculty.olin.edu/∼clee1/)	

## 6 Future Research Directions

In this section, we attempt to predict some future research directions that will be valuable for the examined research fields. First, we anticipate that aerial grasping will be actively pursued in two major directions: 1) analysis, design, modelling, and development of underactuated, adaptive robotic gripper designs and 2) integrating quick-release mechanisms into the grippers. Secondly, we expect that there will be two major future research directions for aerial robots with specific mechanisms that facilitate aerial grasping: 1) analysis, design, modelling, and development of reconfigurable frame designs and 2) integrating appropriate folding robotic arms into aerial robots. Finally, research on aerial robots with perching capabilities is expected to have two major future directions: 1) enhancing the performance of electrostatic adhesives, reducing the size and weight of the battery on board and 2) investigating novel attaching materials that cannot be affected by humidity.
